# Integration of genetic evidence to identify approved drug targets

**DOI:** 10.1186/s13073-026-01689-9

**Published:** 2026-07-06

**Authors:** Samuel Moix, Marie C. Sadler, Zoltán Kutalik

**Affiliations:** 1 Department of Computational Biology, UNIL, Lausanne, 1015 Switzerland; 2 Swiss Institute of Bioinformatics, Lausanne, 1015 Switzerland; 3 University Center for Primary Care and Public Health, Lausanne, 1015 Switzerland

**Keywords:** GWAS, exome, eQTL, pQTL, gene prioritization, drug target discovery, Mendelian randomization, multi-omics integration

## Abstract

**Background:**

Drugs targeting genes supported by human genetic evidence are more likely to succeed in clinical trials. While previous approaches have benchmarked individual methods such as genome-wide association studies (GWAS), rare variant burden testing, and quantitative trait locus (QTL)-informed Mendelian randomization, it remains unclear how best to integrate these signals for drug target discovery.

**Methods:**

We compared gene-prioritization strategies across 30 complex traits, evaluating their ability to recover approved drug targets compiled into lenient and moderate gold-standard sets from six curated databases. Gene-level association scores from GWAS, expression QTL, protein QTL, and exome-based analyses were integrated using five unsupervised approaches. Predictive performance was assessed with area under the receiver operating characteristic curve (AUROC) and enrichment-based statistics.

**Results:**

Across traits, GWAS alone ranked known drug targets on average $\sim$652 ranks (3.42%) above random expectation, and the minimum-rank-based integration strategy further improved performance by approximately $\sim$558 positions (2.93%), achieving the best AUROC in 23 of 30 traits. Genetic correlation and drug target overlap across trait pairs showed a significant positive association ($r = 0.193;\ p = 5.46e{-}5$). Cross-trait analyses further revealed that prioritization scores derived from related diseases could at times equal or even surpass a trait’s own performance. For instance, coronary artery disease data improved the prediction of stroke targets ($p = 0.004$), while inflammatory bowel disease data enhanced the prioritization of chronic kidney disease targets ($p = 0.014$).

**Conclusions:**

These results demonstrate that using the strongest signal from complementary genetic prioritization methods, combined with information from genetically related traits, systematically strengthens drug target identification across complex diseases.

**Supplementary Information:**

The online version contains supplementary material available at 10.1186/s13073-026-01689-9.

## Background

Identifying potential drug targets is a critical early step in the drug development process, with the aim of discovering biological molecules, typically proteins, that play key roles in disease pathways and can be modulated by therapeutic compounds. These targets may include enzymes, receptors, ion channels, or genes whose activity or expression contributes to the onset or progression of a disease. Ultimately, effective drug target discovery increases the likelihood of developing successful therapies, reduces late-stage failures, and contributes to more personalized and efficient treatments for a wide range of diseases. In recent years, the integration of human genetics into this process has gained momentum, as accumulating evidence shows that drugs with strong genetic support are significantly more likely to succeed in clinical trials [1–3]. While caution is needed to avoid confirmation bias, especially when adopting a more permissive view of genetic links (i.e., also considering indirect associations such as related traits), retrospective analyses suggest that nearly two-thirds of FDA-approved drug targets have some prior genetic connection to their indication or a closely related phenotype [4]. This highlights considerable untapped potential for genetically informed drug target discovery. Experimental approaches such as CRISPR/Cas9 or RNA interference screens, and phenotypic screening of compounds in cell lines and organoids, provide direct evidence linking genes to phenotypes in model systems [5, 6]. While essential for validation, these methods often require extensive resources and may lack direct human in vivo relevance. An alternative approach is to improve scalable statistical methods applied to human genetic biobank data. In a previous study [7], we evaluated gene scoring methods applied to genome-wide association studies (GWAS) [8–13], Mendelian randomization (MR) leveraging molecular quantitative trait loci (QTLs) [14, 15], and gene-based burden tests using exome sequencing data [16] individually to assess their effectiveness in directly predicting approved drug targets. However, we observed that top-ranked targets proposed by different approaches have limited predictive ability and showed only moderate overlap, indicating they carry partially orthogonal information. Based on this observation, our next objective is to assess whether integrating evidence from these sources could improve predictive performance for *in silico* target identification using human genetics.

While mapping diseases to genetic variants or genomic regions is relatively straightforward, linking these entities to genes can be a very complex and context-dependent task. Recent approaches increasingly combine multi-omics data to improve variant-to-gene mapping [17]. Frameworks like Open Targets use machine learning to derive locus-to-gene scores by integrating fine-mapping, QTL colocalization, and regulatory annotations [18–20], while PoPS ranks genes based on their functional similarity to polygenic trait patterns [21]. Combining different lines of evidence is not straightforward, and most approaches propose some weighted scores as a priority rank (e.g., Open Targets), but the field lacks a systematic evaluation of integration approaches. To fill this gap, we assess how to combine most efficiently GWAS-based gene scoring, MR with molecular QTLs, and exome-based burden tests to improve the identification of genetically supported drug targets.

## Methods

### Data

#### Approved drug targets

Disease-associated drug target genes were compiled from established public databases, following methods described elsewhere [7]. In summary, drug-disease associations were retrieved from DrugBank [22], Ruiz et al. [23], and ChEMBL [24], while drug-target interactions came from DGIdb [25], STITCH [26], and ChEMBL, forming five database combinations. We then added targets from the Therapeutic Target Database [27] (TTD) as a sixth resource (see Additional file 1: Table S1 for extraction details). While some redundancy exists due to database source overlap (e.g., DGIdb partially aggregates data from TTD), we generated a consensus database of approved drug target genes per disease by applying two filtering strategies based on the number of sources reporting each gene: (i) a lenient approach requiring presence in at least two of the six sources, and (ii) a more moderate approach requiring presence in at least three. A single-source definition was considered insufficiently robust, yielding 3,197 unique target genes. The $\ge$2 threshold retained 2,277 unique target genes across the 30 traits, allowing stable performance estimation and cross-trait comparisons, whereas the $\ge$3 threshold reduced the set to 759 target genes, yielding a cleaner collection more consistent with the expected number of validated human drug targets [28]. At this level, however, target counts per trait already become limited, particularly in analyses restricted to top percentiles, where sparsity reduces resolution to detect nuanced differences between methods. More stringent cutoffs ($\ge$4 or $\ge$5 sources; 583 and 208 genes, respectively) were therefore not pursued. Dataset overlap is shown in Additional file 2: Fig. S1.

Some drugs were linked to pharmacogenes involved in drug metabolism and other pharmacokinetic or regulatory processes, rather than functioning as direct therapeutic targets. Although curated lists of such genes exist (e.g., VIP genes from PharmGKB/ClinPGx [29]), they often include only primary examples (e.g., *CYP3A4*) and may omit related regulators (e.g., *NR1I2*). To further exclude likely non-target pharmacogenes without relying on a specific list, we additionally removed all genes annotated as drug targets for more than 10 distinct traits in the lenient set. This threshold was motivated to ensure the selection of genes targeted by drugs implicated across multiple major ICD categories (as several studied traits shared such categories), thereby aiming to capture broadly acting pharmacogenes. This filtering step excluded 202 genes from the final drug target set (see Additional file 1: Table S2). While this threshold is still somewhat arbitrary, changing it to 8 or 12 did not materially affect the results (see Additional file 2: Fig. S2).

#### Prioritized disease-associated genes

Disease-associated genes were prioritized using four previously described methods [7]: GWAS gene scores from PascalX [8], two molecular QTL-GWAS gene scores leveraging MR (using whole blood expression QTLs from eQTLGen and protein QTLs [30] from the Pharma Proteomics Project [31]), and exome gene scores from gene burden test results [32]. Scores and disease GWAS data were reused from the same study [33–35]. However, while the previous study’s protein QTL-based scores were derived from deCODE [36] protein QTLs, we recomputed these using pQTLs from the UK Biobank’s Pharma Proteomics Project [31]. Mendelian randomization was performed to estimate protein-trait causal effects using cis-pQTLs ($\pm 1$ Mb from gene boundaries) identified in blood plasma ($n = 54,219$). Independent instrumental variables (IVs) ($r^2 < 0.01$) were selected based on strong association with the exposure ($p < 1 \times 10^{-6}$) and clumped using PLINK v1.9 [37] ($p_1 = 0.0001$, $p_2 = 0.01$, $kb = 250$, $r^2 = 0.01$). SNPs (and associated proteins) within the HLA region (chr6:25,000,000–37,000,000; GRCh37/hg19) were removed due to its complex long-range linkage disequilibrium structure [38]. Additional exclusions were applied for allele frequency differences ($\ge 0.05$) between exposure-outcome datasets and Steiger filtering ($Z \le -1.96$) to ensure directionality. After filtering, 2,037 proteins with at least one valid IV remained for analysis. MR was conducted using the TwoSampleMR R package (v0.5.7) [39], primarily through the inverse variance weighted method (see Additional file 1: Table S3).

### Integration strategies

Gene and protein identifiers were harmonized across methods using HGNC symbols from the HUGO Gene Nomenclature Committee. Identifier conversions were performed using biomaRt (version 2.60.1) [40]. *P*-values obtained from different methods were transformed into percentile ranks, where top percentiles indicate stronger associations, and bottom percentiles correspond to unassociated genes. For the GWAS-based method, some *p*-values were below machine precision, resulting in identical values. In such cases, if additional ranking metrics were available, the minimum value among these alternative measures was used as a secondary ranking criterion. Genes with the lowest GWAS *p*-values were then ordered based on this secondary ranking and redistributed along a uniform scale between zero and the minimum GWAS percentile. These adjusted ranks were subsequently converted back into percentiles to ensure meaningful differentiation while preserving their relative order. The final ranking metric was then derived by selecting the minimum percentile across all evaluated methods, followed by rescaling the obtained metric into percentiles (0–100) to obtain the final ranking score.

We further tested four alternative strategies for integrating data across sources. The mean approach computes the arithmetic average of each gene’s available percentile ranks, giving equal weight to all data sources. The Open Targets inspired weighted-sum approach ranks non-missing percentile values across methods and weights them according to their rank, scaling everything by the total of the weights, as given by the following formula. For $i = 1,\dots ,k$ (where $k$ is the number of non-missing values), let the sorted percentiles satisfy

$$\begin{aligned} (100 - x_{(1)}) \;\ge \; (100 - x_{(2)}) \;\ge \; \cdots \;\ge \; (100 - x_{(k)}). \end{aligned}$$

Then

$$\begin{aligned} S \;=\; \frac{\sum \limits _{i=1}^{k} \left( 100 - x_{(i)}\right) \,\frac{1}{i^2}}{\sum \limits _{i=1}^{k} \frac{1}{i^2}}, \qquad \text {combined percentile} \;=\; 100 - S, \end{aligned}$$

where each weight $1/i^2$ gives more influence to top percentiles. The score *S* is then back-transformed to the original scale by computing $100 - S$, which serves as the final combined percentile for ranking. The product approach sums the natural logs of all non-missing percentile ranks and divides by the number of non-missing values (i.e., the log of the geometric mean). The PCA approach begins by converting the resulting *p*-values of each prioritization method to probit z-scores using $z = \Phi ^{-1}(1 - p)$. Infinite values from extremely small *p*-values are clipped to the most extreme finite z-scores, and missing values are imputed using the median z-score for that method. The completed z-score matrix is then centered and scaled, and principal component analysis is applied. The first principal component is used as the new score for each gene, and its sign is flipped if needed to ensure consistency with the mean z-score. As with the minimum-percentile approach, all resulting composite scores (mean, weighted sum, product, and PCA) were rescaled to a common 0–100 percentile scale.

### Assessment of predictive performance

Following previous findings [7], the GWAS-based method generally showed the strongest performance and was therefore used as the baseline for our comparisons. Gene burden tests based on exome data also performed well; comparisons with this method are provided in Additional file 2: Fig. S3. All comparisons were conducted within a matched gene space (e.g., when comparing to GWAS, we included only genes present in that method to ensure fairness).

We used two main strategies to assess drug-target enrichment. First, we evaluated enrichment of drug targets across score percentiles using Fisher’s exact test, reporting odds ratios and 95% confidence intervals for up to the top 5% of genes. Second, we compared the distributions of drug target prioritization scores using one-sided t-tests. For instance, we compared the mean GWAS-based prioritization percentiles of drug targets to those obtained from the integrated approaches. Overall performance was also evaluated using AUROC, computed via the *pROC* package (version 1.18.5) [41].

To assess cross-disease prediction, we used the minimum-based prioritization scores and compared, for all diseases, the mean score of drug targets versus non-targets. We then evaluated whether drug targets for a given disease were better prioritized by their own scores or those obtained for another disease. Specifically, we generated gene ranks based on genetic data obtained for each trait and contrasted these ranks for target and non-target genes for the drug target set of a focal trait. For each drug target set (defined for a focal trait), we picked the trait where the ranks of the targets and non-targets were the most different (in terms of t-test *p*-value). Finally, we compared the best-performing trait’s contrast to that computed from the rankings provided by the focal trait.

To assess whether such cross-disease matches could occur by chance, we constructed an empirical null to obtain a *best-random control*. In each of 100 replicates, we simulated gene-level z-scores for all traits from a multivariate normal distribution with a positive-definite, symmetric covariance matrix derived from the genetic covariance matrix between the respective traits. The simulated scores were then converted to percentiles and tested for the difference between targets and non-targets of the focal trait using a one-sided t-test. For each replicate, we repeated the cross-disease analysis using the random prioritization scores. For each drug-target set, we identified the random profile yielding the most significant *p*-value. These best differences were collected across replicates and tested against an empirical null using a one-sample t-test to assess whether the observed cross-trait enrichments exceeded what would be expected by chance.

### Bending genetic covariance matrix

We constructed a disease-by-disease genetic covariance matrix and a corresponding standard error matrix using pairwise estimates from LDSC [42, 43]. Because heritability estimates can vary slightly across harmonized disease pairs, we filled the diagonals of both matrices with the average heritability values per disease, computed across all pairwise combinations. To ensure that the covariance matrix was positive-definite while accounting for estimate precision, we applied the *bend()* function from the *mbend* R package (version 1.3.1) [44], using a weight matrix defined as the inverse squared standard errors of the respective estimates. Finally, we transformed the bent covariance matrix to the corresponding correlation matrix by rescaling it to unit diagonal (see Additional file 1: Tables S4-S6).

### Cross-trait Mendelian randomization

To assess whether causal relationships between diseases could explain cross-trait prediction patterns, we performed pairwise MR analyses between traits. These analyses followed the general MR framework described above for protein-trait MR (see Methods: “Prioritized disease-associated genes” section), with appropriate modifications to IV selection for complex traits. Specifically, genome-wide significant variants associated with the exposure trait ($p < 5\times 10^{-8}$) were used as IVs. For each trait pair, the MR association testing the effect of one trait as the exposure on another trait as the outcome was compared with the improvement (i.e., mean difference in ranking between drug targets and non-target genes) in cross-trait target prioritization obtained when the exposure trait was used to score genes and prioritize drug targets of the outcome trait. MR significance was then correlated with this improvement (see Additional file 1: Table S7).

## Results

### Methodological overview

**Fig. 1:**
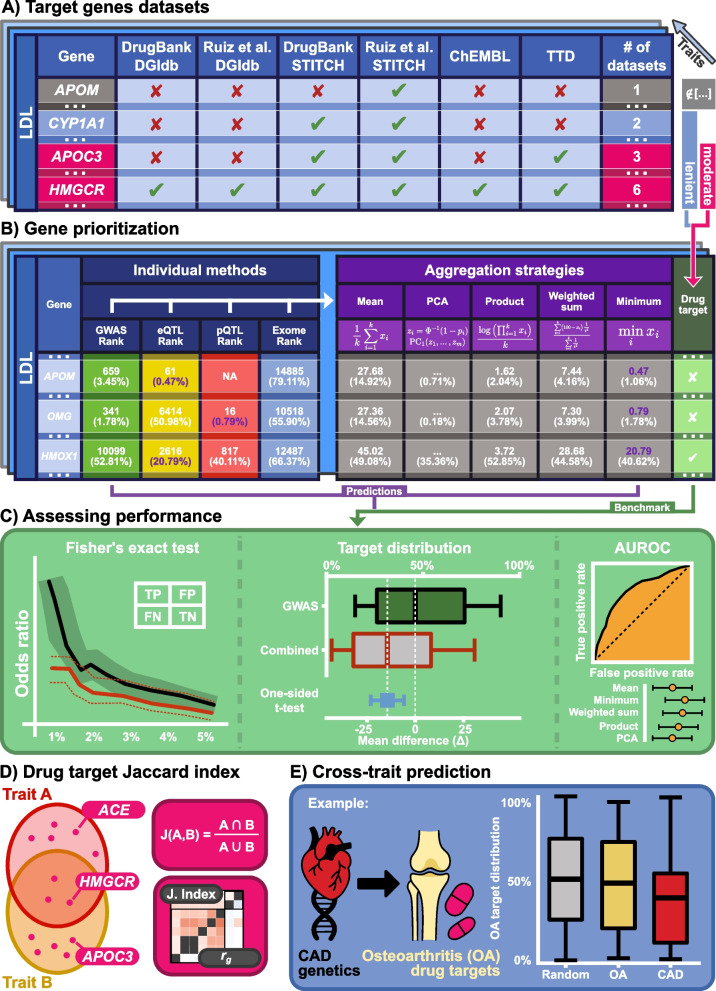
Schematic representation of the study’s workflow. **A** Target gene datasets: The table shows six sources of approved drug target data. For each gene, the number of datasets reporting it as a target is indicated, and two target sets are defined: lenient ($\ge$2 datasets) and moderate ($\ge$3 datasets). Equivalent sets are defined per disease. **B** Gene prioritization: Genes are first ranked independently by multiple genetic evidence sources (GWAS, eQTL, pQTL, and exome sequencing), producing method-specific percentile ranks ($x_i$; 0–100 scale, lower is better). These individual method results are then aggregated using five strategies (mean, PCA, product, weighted sum, and minimum), as indicated by the white arrow. The resulting aggregated scores are re-ranked across all genes using an empirical cumulative distribution function to obtain final percentile scores (shown in parentheses; see Methods: “Integration strategies” section). Percentiles used in the minimum aggregation strategy are highlighted in purple. Drug target evidence for each gene is derived from panel A (blue: lenient; pink: moderate sets) and used for benchmarking. Formulas for each aggregation strategy are shown; here *k* denotes the number of measured individual methods for a gene, *m* the number of genes. **C** Assessing performance: Left; Fisher’s exact test is applied to evaluate enrichment of true drug targets across rank thresholds (TP = true positives, FP = false positives, FN = false negatives, TN = true negatives), reporting the odds ratio across aggregation strategies. Middle; rank distributions are compared using a one-sided t-test to assess performance improvements of the new strategy over standard GWAS. Right; the area under the receiver operating characteristic curve (AUROC) is computed for each strategy per trait. **D** Drug target Jaccard index: shows the overlap of drug targets between datasets using the Jaccard index, which is then compared to genetic correlations ($r_g$). **E** Cross-trait prediction: illustrates the use of genetic information from one trait to predict drug targets of another, exemplified by using coronary artery disease (CAD) gene results to predict osteoarthritis (OA) drug targets. Performance is compared through the distribution of rank-percentiles for drug targets (e.g., OA drug targets) relative to non-target genes

Drug target genes for 30 diseases (see Additional file 1: Table S1) were compiled and classified from six distinct sources (see Methods: “Approved drug targets” section and Additional file 1: Table S8). Based on a consensus strategy, we defined two sets: a lenient one, including genes reported in at least two sources, and a moderate set, requiring agreement across at least three, acknowledging that the selected genes may reflect proteins linked to drug action rather than strictly validated targets. These two gold-standard definitions were selected to balance between robust truth sets and having enough true links to derive meaningful conclusions. For each of the four genetically-informed methods, genes were ranked by *p*-value and converted to rank-percentiles within each disease indication. These methods reflect different assumptions about how genetic variation influences disease: (i) nearby single nucleotide polymorphisms (SNP) may affect gene function (GWAS-based method); (ii) genetic variants may alter gene or protein expression levels (eQTL/pQTL-based methods), and (iii) rare, deleterious mutations may highlight genes with direct functional effects (exome-based method). Aggregation operations, including average, weighted sum, product, and minimum, were applied to rank-percentiles, along with principal component analysis (PCA) applied to transformed *p*-values, followed by rescaling to percentiles for consistency. These aggregation schemes were preferred because they are parameter-free and fully unsupervised, interpretable, and are less subject to overfitting. Performance was evaluated using enrichment odds ratios (OR) to assess signal within top-ranking genes, area under the receiver operating characteristic curve (AUROC), and the distribution of rank-percentiles of true drug targets *vs* non-targets. Next, we explored whether traits with higher genetic correlation are more likely to share drug target genes. In the same vein, we performed cross-trait analyses to assess whether target predictions obtained for one disease could help prioritize targets for a genetically related one. Prioritization was evaluated using the lenient set, as well as a further filtered moderate set to reduce the influence of broadly druggable targets. (Fig. 1).

### Integrating gene priority scores

**Fig. 2:**
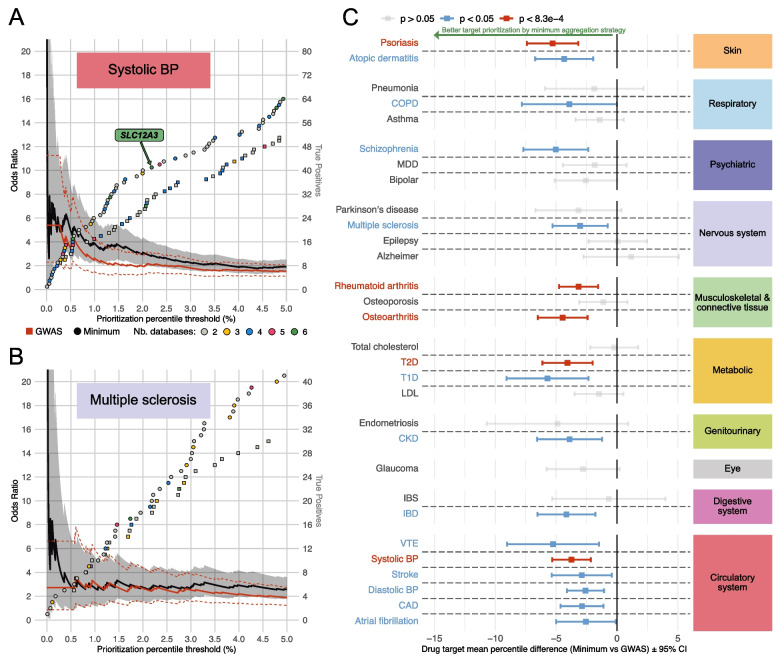
Comparing minimum-based integration to GWAS gene prioritization for drug target identification. **A**–**B** Odds ratios (ORs) for recovering known drug targets among top-ranked genes for *systolic blood pressure* (**A**) and *multiple sclerosis* (**B**) across percentile thresholds (*x*-axis). ORs are shown for the minimum-based (black) and GWAS (red) approaches; shaded areas and dotted lines indicate 95% confidence intervals (CI). Dots represent newly recovered true positive drug targets per threshold (circles: minimum, squares: GWAS), with cumulative counts shown on the right *y*-axis. Dot colors reflect the number of supporting datasets: grey (2), yellow (3), blue (4), magenta (5), and green (6). ORs are based on the lenient drug target definition and the left *y*-axis is truncated at OR = 20. In panel A, we highlight the example discussed in the Results section of the true positive SBP target *SLC12A3*, ranked at 2.19% by the minimum-based strategy (not labeled for GWAS as it ranks at 9.80%); at this threshold the enrichment of drug targets corresponds to OR = 2.88 [95% CI 2.02 – 4.03], based on 41 true positive targets. **C** Mean difference between gene prioritization percentiles using the minimum-based versus standard GWAS approach across drug target genes within the GWAS gene space. The *x*-axis shows the mean difference (minimum − GWAS) with 95% confidence intervals (CI). One-sided t-test; CI shown symmetrically for visualization. Negative values indicate improved rankings by the minimum-based strategy. Square dots represent results from the lenient drug target set (genes supported by $\ge 2$ datasets) and their color indicates significance of the difference: grey for non-significant, blue for nominal ($p < 0.05$), and red for Bonferroni significant ($p < 0.05 / 60 = 8.3 \times 10^{-4}$). Diseases are grouped by ICD-10 categories shown in the right boxes

Among the aggregation strategies evaluated in this study, the minimum-based approach generally performed best for prioritizing drug targets across the assessed traits. This behavior can be illustrated with two well-established drug–target examples. Hydrochlorothiazide, a thiazide diuretic used to treat hypertension, acts by inhibiting the sodium–chloride cotransporter encoded by *SLC12A3* [45]. Considering evidence derived from the systolic blood pressure (SBP) phenotype, individual genetic methods rank *SLC12A3* quite differently: GWAS places it at 9.80%, blood eQTL-based MR at 88.0%, and exome burden testing at 0.94%, while the relevant protein is not measured in the available UK Biobank proteomics dataset. When aggregating evidence, the minimum-rank strategy captures the strong burden signal and places *SLC12A3* at 2.19% after re-ranking across all genes (Fig. 2A). Other aggregation strategies prioritize it less strongly: weighted sum 6.62%, product 4.98%, PCA 27.21%, and mean 21.26%. A similar pattern is observed for methotrexate, indicated for rheumatoid arthritis, which acts by inhibiting the enzyme encoded by *DHFR* [46]. In this case, the burden test again provides strong support for *DHFR* (0.20%), whereas eQTL-based MR ranks it at 12.4% and GWAS at 58.2%, with no pQTL measurement available. After aggregation, the minimum-rank strategy prioritizes *DHFR* at 0.86%, compared with 3.34% for the weighted sum, 2.15% for the product strategy, 18.31% for PCA, and 10.31% for the mean approach.

To assess performance across traits in a setting more relevant to practical target prioritization, where only a limited number of candidates would typically be followed up using additional data and experimental approaches, we evaluated enrichment of known drug targets among top-ranked genes across ranking thresholds up to 5%. Odds ratios were calculated across these thresholds for each approach using the lenient benchmark set to maximize the number of discoverable targets. Because GWAS-based prioritization performed on average best among the individual non-aggregated methods, it was used as the primary reference when evaluating improvements obtained through evidence aggregation. Across many traits, GWAS-based prioritization showed considerable overlap with the minimum-based approach, whose enrichment ratio curves frequently intersect (Additional file 2: Figs. S4-S5). While these results show that performance advantage is often sensitive to threshold choice, the minimum-based approach may provide higher enrichment for certain traits, such as SBP and multiple sclerosis (MS). Specifically, the minimum-based strategy yielded higher enrichment than GWAS alone for SBP at the top 1 percentile (OR 3.87 [95% CI 2.40 – 6.01] vs 2.64 [95% CI 1.49 – 4.38]). Moreover, combining information can help disentangle tied top ranks by GWAS (a limitation of that method), as seen for MS at the 0.1 percentile (OR 6.14 [95% CI 0.69 – 25.82] vs 2.72 [95% CI 0.86 – 6.62]). As we move beyond the top 0.5% ranking genes, the differences become even more nuanced, and as we approach 5% enrichment, ORs converge to similar values (OR 1.91 [95% CI 1.43 – 2.49] vs 1.53 [95% CI 1.12 – 2.05] for SBP, and 2.57 [95% CI 1.80 – 3.60] vs 1.87 [95% CI 1.23 – 2.74] for MS). Although these differences are not statistically significant, at the 5% threshold, integration still yields 13 additional true positive targets for SBP and 11 for MS compared to prioritization based only on GWAS, out of 745 and 355 lenient targets (Fig. 2A-B).

To obtain a more general view of prioritization performance across traits, we next examined the distribution of rank percentiles assigned to known drug targets. As a reference point, we first assessed how the GWAS-based approach performs on its own. Compared to random target prioritization (i.e., the 50th percentile), GWAS improved prioritization by an average of 3.42 and 5.34 percentile points in the lenient and moderate drug target set, respectively, translating to rank improvements of $\sim$652 and $\sim$1,018 out of $\sim$19,100 genes. The minimum-based strategy ranked drug targets on average (across traits) 2.93 percentile points higher ($\sim$558 ranks) than the GWAS-based approach, indicating better prioritization (2.46% / $\sim$469 ranks in the moderate set). This improvement was at least nominally significant for 17 of the 30 diseases tested (7 in the moderate subset), and for none of the traits did the minimum rank show worse performance than GWAS (Fig. 2C and Additional file 2: Fig. S6A). The weighted-sum approach also performed well, though slightly less effectively, achieving an average improvement of 1.92 percentile points ($\sim$366 ranks) over GWAS (1.44% / $\sim$274 ranks in the moderate set). In contrast, the PCA-based strategy ranked drug targets on average 0.44 percentile points ($\sim$84 ranks) worse than GWAS, the mean-based approach 0.72 points worse ($\sim$137 ranks), and the product-based strategy achieved only a modest improvement by 0.57 percent ($\sim$109 ranks). These alternative strategies also included cases where performance was nominally worse than GWAS (Additional file 1: Tables S9-S14; Additional file 2: Fig. S6B-E). Although AUROC comparisons revealed a suggestively significant improvement only for psoriasis ($p = 0.029$), the minimum-based strategy achieved the highest AUROC of all tested approaches in 23 of 30 diseases (9 of 30 in the moderate set). Notably, it also yielded the highest average AUROC across traits, with a mean of 0.551 in the lenient set and 0.565 in the moderate set, highlighting its consistent performance across conditions. Compared to GWAS, using the lenient target set, the minimum-based integration showed a slight but significant average improvement ($\Delta = 0.016, p = 0.021$, one-sided t-test), while performance in the moderate set was comparable ($\Delta = 0.011, p = 0.247$; Additional file 1: Tables S15-S16; Additional file 2: Fig. S7).

### Cross-trait prediction

**Fig. 3:**
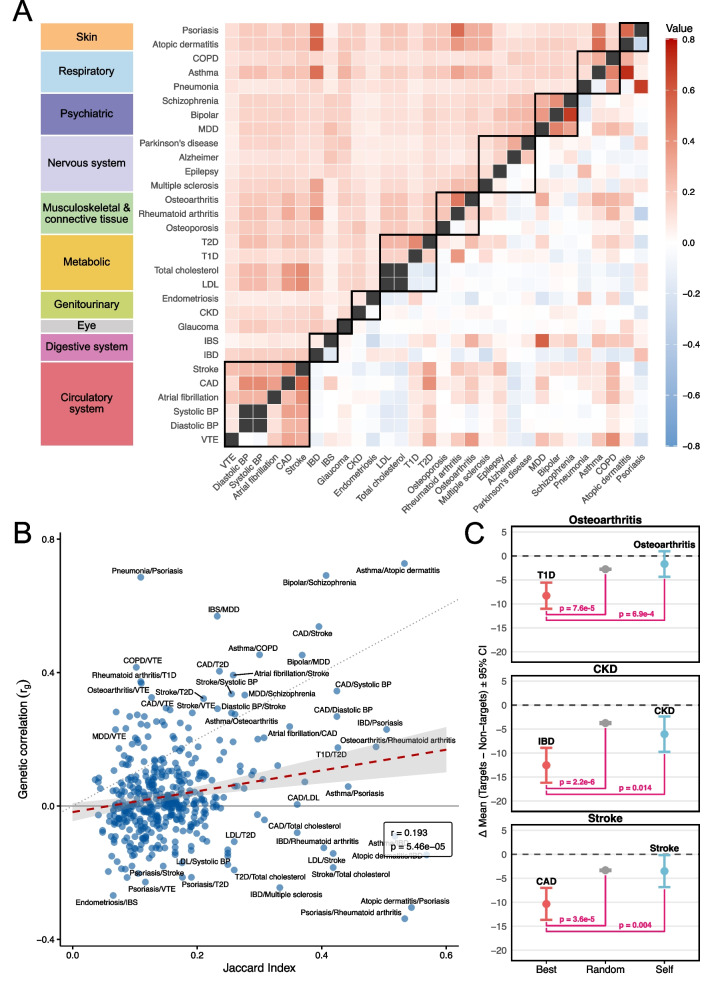
Drug target overlap and cross-trait prioritization. **A** Heatmap comparing drug target genes overlap (upper triangle) and genetic correlation (lower triangle) between disease pairs. Jaccard indices quantify the overlap of drug target genes from the lenient set (supported by $\ge$2 datasets). Genetic correlations ($r_g$) were computed using LDSC [42, 43] based on GWAS summary statistics. Positive $r_g$ values are shown in red, negative in blue, and Jaccard indices are shaded according to the scale on the right. Traits are grouped by ICD-10 categories, indicated on the left *y*-axis and emphasized with black borders along the diagonal. **B** Scatter plot comparing genetic correlation (*y*-axis) to drug target overlap (Jaccard index, *x*-axis) for all disease pairs (blue circles). A red dashed line represents the linear regression fit with 95% confidence interval in grey shading. The dotted diagonal is the identity line, and the solid horizontal grey line marks zero genetic correlation. Regression estimates are displayed in the bottom-right box. **C** Illustrative examples of cross-trait prediction of drug targets. For three diseases – osteoarthritis (top), chronic kidney disease (middle), and stroke (bottom) – we compare the mean percentile difference between drug targets and non-targets based on the minimum-based strategy. More negative values indicate better prioritization of true drug targets. The *x*-axis shows three predictor conditions: the best-performing external trait (“Best”, red dot), a *best-random control* (“Random”, grey dot; see Methods: “Assessment of predictive performance” section), and the trait itself (“Self”, blue dot). Error bars show 95% confidence intervals. The dotted horizontal line at zero indicates no difference in mean percentiles between targets and non-targets. In pink, *p*-values are reported for the statistical comparison between “Best” and “Random” as well as between “Best” and “Self” predictors

Next, we assessed the overlap of drug targets across diseases to examine the commonalities between traits. For the lenient set, the mean Jaccard index was 0.159 overall and 0.248 within ICD-10 classes, with psychiatric traits showing the highest within-class overlap (0.351, Fig. 3A). For the moderate set, the mean Jaccard index was 0.122 overall and 0.218 within ICD-10 classes, with psychiatric traits again highest (0.468). To account for potential bias from recurrent drug targets, we excluded widespread pharmacogenes and targets implicated in more than ten diseases (see Additional file 1: Table S2), hereafter referred to as very important pharmacogenes (VIP). Across traits, the most recurrent targets correspond to cytochrome P450 drug-metabolizing enzymes (e.g., CYP3A4, CYP2C9, and CYP2D6), reflecting their central role in drug metabolism rather than disease-specific biology. After VIP removal, the mean overlap was 0.071 (lenient) and 0.037 (moderate) overall, and 0.161 (lenient) and 0.128 (moderate) within ICD-10 classes. The more specific the target set was defined, the more striking the increase was for within ICD-10 categories. Across all analyses, diseases within the same ICD-10 category consistently shared more drug targets and displayed higher genetic correlations, consistent with the expectation that biologically related traits are treated by similar therapeutic mechanisms (Additional file 1: Tables S17-S18; Additional file 2: Fig. S8A). To validate this further, we compared drug target overlap between diseases with their genetic correlation, resulting in a correlation coefficient of $r = 0.193$ ($p = 5.46e{-}5$) for the lenient set (Fig. 3B) and $r = 0.265$ ($p = 2.05e{-}8$) for the moderate set with VIP included. When removing VIP, we obtained $r = 0.188$ ($p = 8.28e{-}5$) for the lenient set and $r = 0.296$ ($p = 3.25e{-}10$) with the moderate set (Additional file 2: Fig. S8B).

Given this concordance, we further evaluated the effectiveness of cross-trait drug target prioritization, determining whether prioritization scores from one disease could predict targets in another. To this end, for each disease, we evaluated three strategies: self-prioritization, best cross-trait prioritization (i.e., the trait yielding the largest improvement in percentile ranks between targets and non-targets), and a *best-random control* derived from an empirical null (see Methods: “Assessment of predictive performance” section). We conducted these analyses in a relaxed manner, using the lenient target set that included VIP genes, and in a more stringent manner, using the moderate set of targets with VIP genes excluded. In both cases, for all diseases, we identified at least one non-target disease whose prioritization performed comparably to the disease’s own (Additional file 2: Figs. S9-S12).

Notably, with the lenient set, osteoarthritis targets were significantly better prioritized using type 1 diabetes (T1D) scores ($p = 6.9e{-}4$), which also significantly outperformed the *best-random control* ($p = 7.6e{-}5$). Similarly, chronic kidney disease (CKD) targets were better predicted using inflammatory bowel disease (IBD) gene ranks ($p = 0.014$), with performance exceeding that of the *best-random control* ($p = 2.2e{-}6$). For closely related traits such as coronary artery disease (CAD) and stroke, cross-trait prioritization proved robust, with CAD significantly improving stroke target prediction ($p = 0.004$) and outperforming the *best-random control* ($p = 3.6e{-}5$, Fig. 3C). In the stringent setup, less overlap was observed across configurations, but CAD still emerged as the best predictor trait for stroke. While it did not significantly improve stroke target prediction ($p = 0.135$), it still outperformed the *best-random control* ($p = 0.007$), in line with the idea that genetically related traits can improve drug target prioritization through leveraging shared biological mechanisms (Additional file 2: Fig. S8C).

As some of the traits that best predicted targets for another disease showed higher SNP-heritability than the predicted trait itself (e.g., T1D $h^2 = 0.224$ versus osteoarthritis $h^2 = 0.024$), we assessed whether this pattern reflected a broader trend. Across all traits, however, we did not observe a relationship between trait heritability and improvements in cross-trait target prioritization in either configuration, suggesting that differences in heritability alone do not explain the observed cross-trait prediction performance (Additional file 2: Fig. S13).

We also investigated whether causal relationships between diseases could contribute to the observed cross-trait predictive power. To do so, we performed pairwise MR analyses across traits and assessed whether the strength of causal evidence between diseases was associated with improvements in cross-trait target prediction. In both configurations, we observed a modest but significant correlation (lenient: $r = -0.12, p = 1.2e{-}3$; moderate without VIP: $r = -0.10, p = 8.3e{-}3$), indicating that predicted drug targets for traits with evidence of causal influence on a downstream disease tend to have the potential to improve overlap with the downstream trait’s targets (i.e., more negative mean percentile differences between drug targets and non-targets; Additional file 2: Fig. S14).

### Bias from limited protein data

Given substantial missing protein measurement data, we first evaluated whether these proteins were enriched as drug targets. Proteins measured in the Pharma Proteomics Project set showed significant enrichment (Fisher’s exact test: lenient set $OR = 3.79, p = 3.97e{-}130$; moderate set $OR = 3.39, p = 1.31e{-}44$). We repeated our analyses on a restricted set of $\sim$1,360 protein-coding genes for which ranks could be established by all four methods. When examining the rank-percentile distribution of drug targets, no significant changes were observed across traits. The minimum-based strategy ranked targets, on average, 0.943 percentile points worse than GWAS in the lenient set with suggestive evidence of improvement for epilepsy only ($p = 0.022$), but showed an improvement of 1.489 percentile points in the moderate set. The weighted-sum approach also performed slightly worse by 1.079 percentile points in the lenient set, improving marginally by 0.890 percentile points in the moderate set, showing nominal improvements for rheumatoid arthritis and epilepsy. Similarly, the product-based strategy performed 0.955 points worse in the lenient set but improved by 0.520 points in the moderate set. The mean-based and PCA-based strategies showed consistently worse performance compared to GWAS alone (mean: 1.483 points worse in lenient, 1.750 points worse in moderate; PCA: 1.097 points worse in lenient, 0.637 points worse in moderate; Additional file 2: Fig. S15). Due to the limited size of the subset, meaningful comparisons of OR within the top 5% were challenging, averaging only five targets in the lenient set and three in the moderate set (Additional file 2: Figs. S16-S17). AUROC analyses showed comparable performance across strategies, with GWAS alone achieving the highest AUROC in 15 out of 30 traits in the lenient set, followed by the minimum strategy in five traits. In the moderate set, GWAS alone achieved the highest AUROC in 11 of 29 traits (excluding IBS, without targets in the set), with the minimum strategy ranking highest in nine traits (Additional file 1: Tables S19-S20; Additional file 2: Fig. S18). Overall, the minimum-based approach consistently outperformed other combining strategies, although GWAS alone remained highly competitive.

Another way to test whether data from the Pharma Proteomics Project would bias results was to remove the pQTL analysis entirely and only consider the remaining three methods. Doing so again highlighted the advantage of the minimum-based approach, which improved over GWAS by an average of 1.808 percentile points in the lenient set and 1.472 in the moderate set, followed by the weighted-sum strategy with smaller gains (0.921 in lenient and 0.489 in moderate). The observed patterns also suggest that incorporating pQTL information adds value to prediction beyond adding scores for a drug-target-enriched protein set (Additional file 2: Fig. S19).

## Discussion

In this study, we explored how genetically informed drug target prediction methods can be combined to further improve target identification. In particular, we focused on (i) combining methods and (ii) linking traits. To address the first objective, we assessed five statistical approaches for integrating rank-percentiles derived from *p*-values across four genetically-informed methods. Our results indicate that the minimum-based approach consistently outperforms alternative strategies in predicting known targets. For the second goal of this paper, we identified several cases where cross-trait prioritization either exceeded or matched the performance of within-trait target prediction, while still outperforming the (random trait-based) control predictor used as reference. These findings highlight the potential of genetically related traits to improve target identification across diseases.

While leading efforts such as the Open Targets Platform integrate diverse types of evidence for target prioritization, they differ widely depending on the focal trait. Also, using a user-defined weighted sum of harmonic means assumes prior knowledge of which resources are high-confidence, thereby adding an extra component to the prediction and therefore hindering the isolation of the effect of the data integration strategy itself. To this end, we evaluated aggregation strategies of diverse genetics-based evidence under matched data conditions rather than contrasting different data platforms. Within this framework, the minimum-based strategy offers a distinct advantage by highlighting genes supported by at least one method, helping to address limitations related to power, context, or data availability. Indeed, some of the prioritization approaches may lack power for certain genes (e.g., that lack e/pQTLs) or use data from an irrelevant tissue or miss longer-range regulatory mechanisms (GWAS), so it is unlikely that all methods would confirm the involvement of the same gene. Thus, a strong signal obtained by one method should be sufficient evidence, which is reflected in the minimum-rank approach. On the other hand, weaker signals consistently present across methods may be missed. This illustrates a trade-off between certainty and discoverability: the minimum strategy maximizes recall by reducing false negatives, whereas a consensus-based approach increases precision by requiring agreement but at the cost of more false negatives. In short, the minimum and weighted approaches are more sensitive to strong positive outliers, capturing genes supported by a strong signal from any individual source. The product-based aggregation favors strong signals but is sensitive to missing evidence, as weak support from one source can substantially reduce the combined score. In contrast, the mean and PCA behave as consensus approaches, prioritizing genes that show consistent evidence across multiple sources. Our results show that favoring discoverability through the minimum strategy provides a beneficial balance in the context of identifying known approved targets. In practice, candidate genes would require follow-up with additional and orthogonal sources of evidence beyond the methods evaluated in this study.

Due to limited statistical power (given the low number of gold standard targets), the improvement of the minimum-based approach compared to GWAS alone was not statistically significant in terms of enrichment of drug targets in the top 5% prioritized genes. Nevertheless, it was the only aggregation strategy that consistently performed on par with, and in some cases outperformed, GWAS. This pattern was also reflected across the other performance metrics, where the minimum approach repeatedly ranked among the best-performing aggregation strategies. When looking at the global prioritization performance, although percentile differences appear modest, the gain from minimum-based aggregation over GWAS is comparable to the gain of GWAS over random expectation when considering target distribution across all rank percentiles.

Beyond combining individual sources, cross-trait target prioritization represents a complementary strategy. However, it remains challenging to explain why certain cross-trait combinations improve prioritization or to identify what makes a cross-trait relationship informative for target discovery. Among the most notable examples, successful cross-trait predictions arise from diverse biological relationships, including T1D and osteoarthritis, where links appear more indirect [47], IBD and CKD, where evidence supports a moderate connection [48], and CAD and stroke, which show a strong, near-identical vascular relationship. This spectrum highlights that cross-trait relationships stem from a range of biological connections. Although higher trait heritability in these cases might appear as a plausible explanation for improved prioritization performance, we did not observe a consistent trend across all traits. In contrast, traits that are genetically related appear more informative, and a similar pattern emerges when examining causal relationships between traits using MR. Taken together, these observations suggest that traits with high genetic correlation or those involved in shared causal pathways may represent promising candidates for cross-trait target prioritization, although further work will be required to systematically evaluate this hypothesis.

Our study has several limitations: 1) Although genome-wide data are available for most human coding genes, coverage remains limited for proteomics (pQTLs) and somewhat restricted for eQTLs (for tissues with low sample size). This uneven data availability may introduce bias due to the non-random distribution of missingness across methods. Still, the minimum-based approach performs best even without pQTL data, suggesting robustness. As datasets continue to expand and more genes become testable by each approach, this ascertainment bias is likely to decrease. 2) Another key limitation of this study lies in the difficulty of defining a clean and reliable set of approved drug targets. We attempted to address this by focusing on consensus targets reported in multiple databases. However, overlap between sources may in some cases reflect shared annotations/resources rather than truly independent evidence. Moreover, despite efforts to curate the dataset, errors introduced during initial database compilation persist, such as targets linked through combination therapies rather than direct drug effects. While a more stringent curation could reduce noise, it would result in very limited target sets, making, in particular, cross-trait comparisons difficult or uninformative. Our approach reflects a necessary trade-off between gold standard reliability and its completeness, with thresholds chosen to balance signal quality and comparability across traits. 3) A further limitation is that we do not separate drugs intended to modify disease biology from those used mainly for symptomatic relief. For example, we observe cross-prediction with osteoarthritis, where current therapies primarily address symptoms rather than underlying mechanisms, which may bias our overlap estimates. This highlights the need for caution in interpreting such results, and future work may benefit from distinguishing symptomatic from disease-modifying targets, although this requires detailed, disease-specific knowledge of therapeutic action.

## Conclusions

Overall, our results show that combining different types of genetic evidence can help identify the most promising genes for developing new drugs. When evaluating the potential of one focal target, the aim is to gather multiple lines of evidence in support. However, when the aim is to prioritize targets among many, even strong evidence from just one genetic approach may already be enough to highlight a gene as worthy of further study. In our analyses, the most effective strategy was usually to retain genes that showed a strong signal by at least one method, rather than requiring multiple methods to agree, which could overlook potentially important targets. We also found that genetic evidence from related diseases can provide valuable additional clues for identifying promising drug targets.

## Supplementary Information

Additional file 1: Supplementary Tables. Table S1: GWAS datasets and trait definitions. Table S2: Important pharmacogenes. Table S3: Gene-disease association *p*-values across methods. Table S4: Bent genetic covariance matrix. Table S5: Genetic covariance standard errors. Table S6: Trait-level heritability estimates. Table S7: Mendelian randomization estimates. Table S8: Drug target gene annotations across databases. Table S9: Minimum vs GWAS rank differences. Table S10: Weighted-sum vs GWAS rank differences. Table S11: Product vs GWAS rank differences. Table S12: PCA vs GWAS rank differences. Table S13: Mean vs GWAS rank differences. Table S14: Minimum vs exome rank differences. Table S15: AUROC performance across strategies (lenient set). Table S16: AUROC performance across strategies (moderate set). Table S17: Genetic correlation and target overlap (lenient set). Table S18: Genetic correlation and target overlap (moderate set). Table S19: AUROC performance across strategies (lenient set, complete data). Table S20: AUROC performance across strategies (moderate set, complete data).

Additional file 2: Supplementary Figures. Fig. S1: Drug target overlap across datasets. Fig. S2: Robustness of cross-trait prediction results to the pharmacogene (VIP) exclusion threshold. Fig. S3: Comparing minimum-based integration to exome gene prioritization for drug target identification. Fig. S4: Recovery of known drug targets in the lenient set across top five percentiles. Fig. S5: Recovery of known drug targets in the moderate set across top five percentiles. Fig. S6: Comparing integration strategies to GWAS gene prioritization for drug target identification. Fig. S7: AUROC performance comparison across strategies and drug target sets. Fig. S8: Drug target overlap and cross-trait prioritization without VIP genes. Fig. S9: Cross-trait drug target prediction from the lenient set (supported by $\ge$2 datasets). Fig. S10: Cross-trait drug target prediction from the moderate set (supported by $\ge$3 datasets) without VIP genes. Fig. S11: Best cross-trait prediction of drug targets with random baseline comparison from the lenient set. Fig. S12: Best cross-trait prediction of drug targets with random baseline comparison from the moderate set without VIP genes. Fig. S13: Relationship between SNP-heritability and cross-trait target prioritization performance. Fig. S14: Relationship between MR evidence across traits and cross-trait target prioritization performance. Fig. S15: Comparing integration strategies to GWAS gene prioritization for drug target identification on complete data. Fig. S16: Recovery of known drug targets in the lenient set across top five percentiles on complete data. Fig. S17: Recovery of known drug targets in the moderate set across top five percentiles on complete data. Fig. S18: AUROC performance comparison across strategies and drug target sets on complete data. Fig. S19: Comparing integration strategies to GWAS gene prioritization for drug target identification without pQTL results.

## Data Availability

This paper analyzes publicly available data, as presented in the previous publication by Sadler et al. [7] and as described in the Methods. This includes pQTL data from Sun et al. (http://ukb-ppp.gwas.eu), exome-based gene scores from Backman et al., and eQTL data from Võsa et al. (https://www.eqtlgen.org/cis-eqtls.html). GWAS summary statistics were primarily derived from the Pan-UK Biobank resource (https://pan.ukbb.broadinstitute.org/), with specific datasets also obtained from the Neale Lab (https://www.nealelab.is/uk-biobank), Han et al. [35], and the GWAS Catalog (https://www.ebi.ac.uk/gwas/). Original drug target and indication datasets were obtained from DrugBank (https://go.drugbank.com/), ChEMBL (https://www.ebi.ac.uk/chembl/), DGIdb (https://www.dgidb.org/), STITCH (https://stitch-db.org/), and the Therapeutic Target Database (TTD; https://db.idrblab.net/ttd/). Lists of pharmacogenes were obtained from ClinPGx (https://www.clinpgx.org/). For details such as referenced studies and drug target lists, see Additional file 1. Program code generated in this study is available under the Creative Commons Attribution 4.0 International License (CC BY 4.0) on GitHub (https://github.com/cChiiper/UNIL_SGG_DrugTarget) [49].

## Abbreviations

AUROC: Area under the receiver operating characteristic curve
CAD: Coronary artery disease
ChEMBL: ChEMBL database
CI: Confidence interval
CKD: Chronic kidney disease
DBP: Diastolic blood pressure
DGIdb: Drug Gene Interaction database
eQTL: Expression quantitative trait locus
FDA: Food and Drug Administration
FN: False negative
FP: False positive
GWAS: Genome-wide association study
HGNC: HUGO Gene Nomenclature Committee
HLA: Human leukocyte antigen
IBD: Inflammatory bowel disease
IBS: Irritable bowel syndrome
ICD-10: International Classification of Diseases, 10th Revision
IV: Instrumental variable
LDSC: Linkage disequilibrium score regression
LDL: Low-density lipoprotein
MR: Mendelian randomization
MS: Multiple sclerosis
OA: Osteoarthritis
OR: Odds ratio
PCA: Principal component analysis
pQTL: Protein quantitative trait locus
QTL: Quantitative trait locus
SBP: Systolic blood pressure
SNP: Single nucleotide polymorphism
STITCH: Search tool for interactions of chemicals
T1D: Type 1 diabetes
TN: True negative
TP: True positive
TTD: Therapeutic Target Database
VIP: Very important pharmacogene
