# Supplementary material for: Integration of genetic evidence to identify approved drug targets
Source: Genome Med. 2026 Jul 6;18:98. doi: 10.1186/s13073-026-01689-9 (PMC13335195; doi:10.1186/s13073-026-01689-9)
Supplement: Supplementary file 2 — Additional file 2: Supplementary Figures. Fig. S1: Drug target overlap across datasets. Fig. S2: Robustness of cross-trait prediction results to the pharmacogene (VIP) exclusion threshold. Fig. S3: Comparing minimum-based integration to exome gene prioritization for drug target identification. Fig. S4: Recovery of known drug targets in the lenient set across top five percentiles. Fig. S5: Recovery of known drug targets in the moderate set across top five percentiles. Fig. S6: Comparing integration strategies to GWAS gene prioritization for drug target identification. Fig. S7: AUROC performance comparison across strategies and drug target sets. Fig. S8: Drug target overlap and cross-trait prioritization without VIP genes. Fig. S9: Cross-trait drug target prediction from the lenient set (supported by $\ge$2 datasets). Fig. S10: Cross-trait drug target prediction from the moderate set (supported by $\ge$3 datasets) without VIP genes. Fig. S11: Best cross-trait prediction of drug targets with random baseline comparison from the lenient set. Fig. S12: Best cross-trait prediction of drug targets with random baseline comparison from the moderate set without VIP genes. Fig. S13: Relationship between SNP-heritability and cross-trait target prioritization performance. Fig. S14: Relationship between MR evidence across traits and cross-trait target prioritization performance. Fig. S15: Comparing integration strategies to GWAS gene prioritization for drug target identification on complete data. Fig [file 13073_2026_1689_MOESM2_ESM.pdf]

Integration of genetic evidence to identify approved drug targets

Samuel Moix[1,2,*], Marie C Sadler[1,2,3], and Zoltán Kutalik[1,2,3,*]

[1]Department of Computational Biology, UNIL, Lausanne 1015, Switzerland

[2]Swiss Institute of Bioinformatics, Lausanne 1015, Switzerland

[3]University Center for Primary Care and Public Health, Lausanne 1015, Switzerland

[*]Corresponding authors

Additional file 2: Supplementary Figures

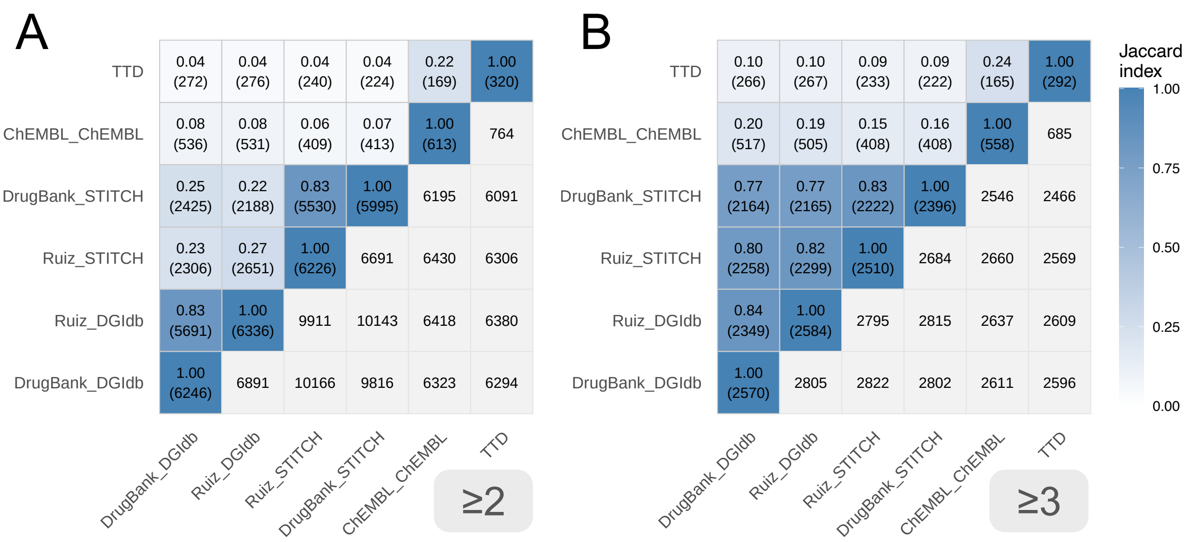

Fig. S1: Drug target overlap across datasets. Heatmaps showing the overlap of drug targets across six different datasets (TTD: Therapeutic Target Database, ChEMBL, DrugBank, Ruiz *et al*, DGidb: Drug Gene Interaction Database), evaluated across 30 diseases (i.e., shared targets across diseases are counted multiple times). The upper triangle and diagonal of each matrix display the Jaccard index, quantifying the proportion of shared targets between dataset pairs, and are color-coded according to the legend. The corresponding values in parentheses indicate the number of intersecting drug targets. The lower triangle shows the number of drug targets in the union of the two datasets (in grey tiles). **A** shows results for the lenient set (targets appearing in ≥ 2 datasets), while **B** represents the moderate set (targets appearing in ≥ 3 datasets).

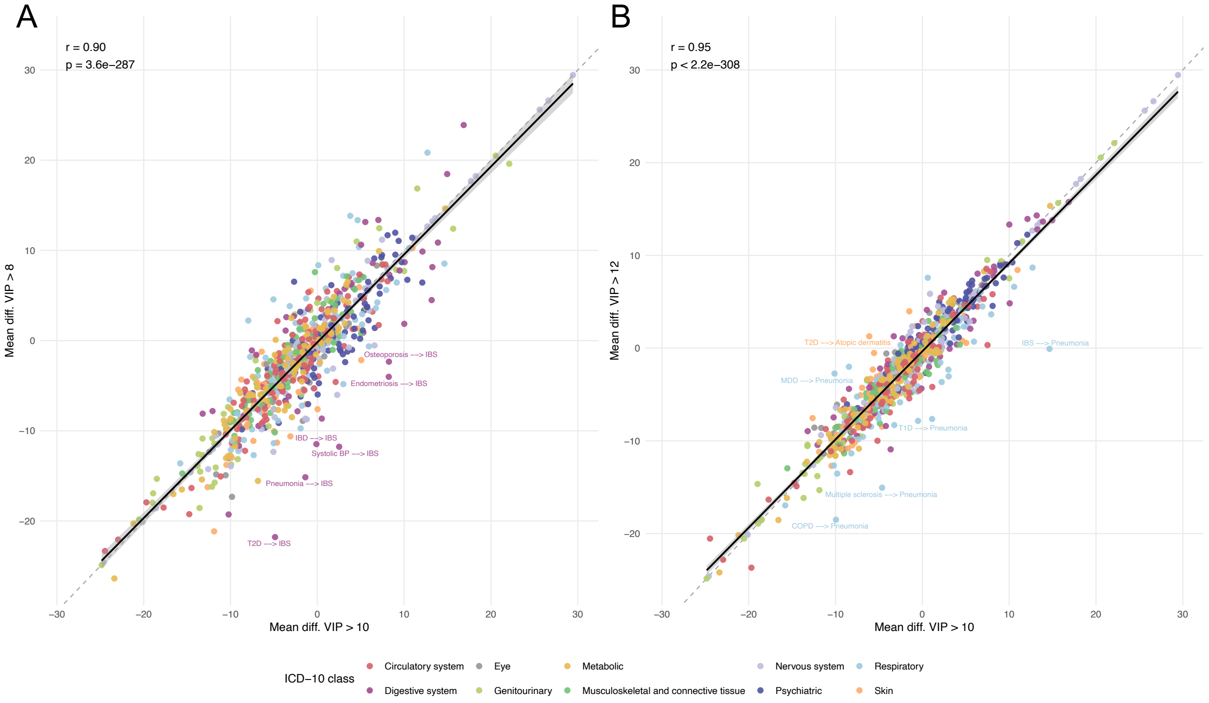

Fig. S2: Robustness of cross-trait prediction results to the pharmacogene (VIP) exclusion threshold. **(A–B)** Comparison of mean differences in target ranking between drug targets and non-targets across all trait pairs when defining pharmacogenes as genes annotated as drug targets for more than 8 **(A)** or more than 12 **(B)** distinct traits, compared to the main analysis threshold (>10 traits; see Additional file 2: Fig. S10). Each point represents a trait pair (score trait → drug target trait), colored by ICD-10 disease category of the drug target trait. The dashed line indicates the identity line, and the solid line shows the linear regression fit with 95% confidence interval in grey shading. Pearson correlation coefficient and corresponding p-value are shown in the top left corner of each panel.

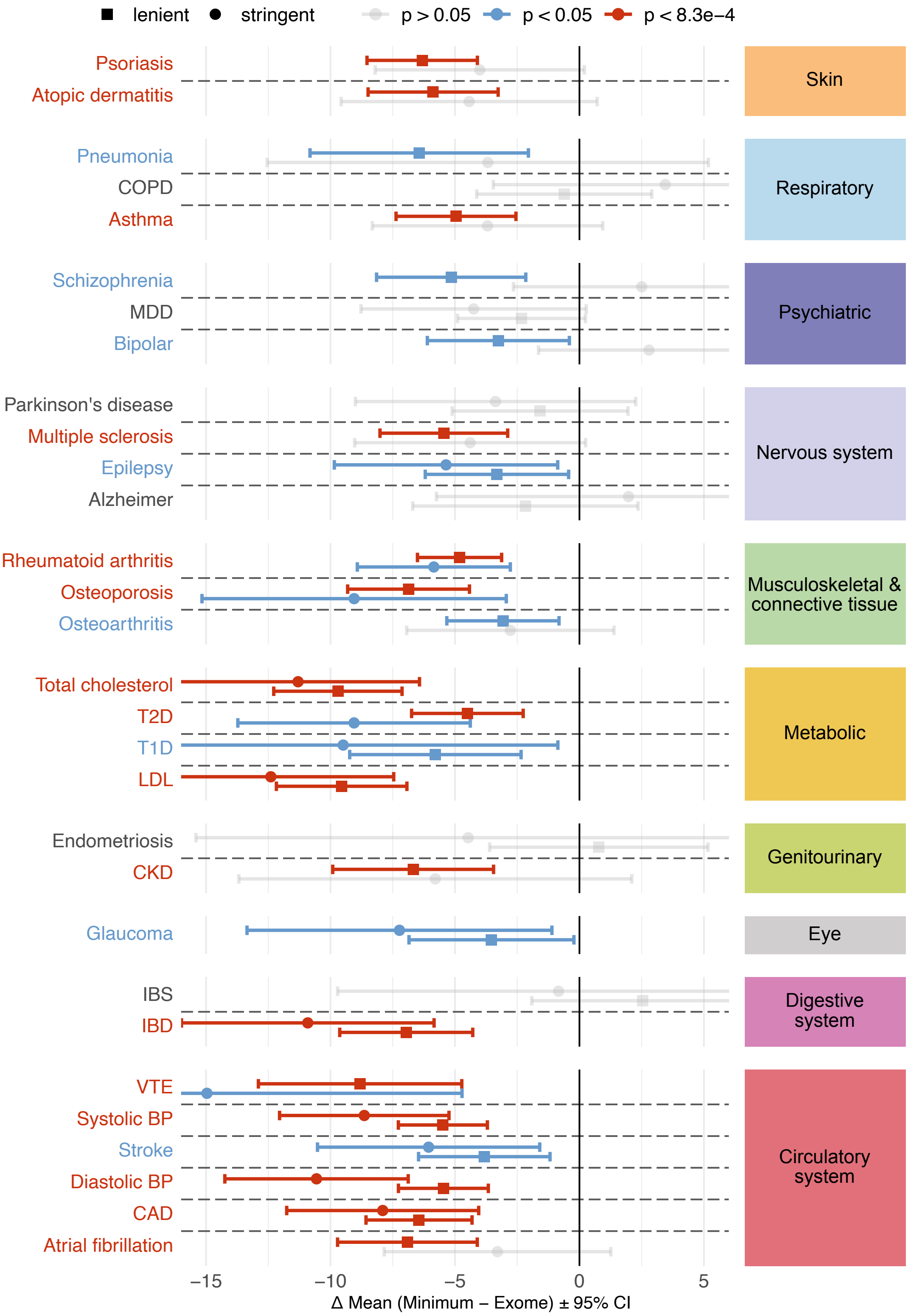

Fig. S3: Comparing minimum-based integration to exome gene prioritization for drug target identification. Mean difference between gene prioritization percentiles using the minimum-based versus standard exome approach, across drug target genes within the exome gene space.

Fig. S3: Comparing minimum-based integration to exome gene prioritization for drug target identification (continued). The x-axis shows the mean difference (minimum − exome) with 95% confidence intervals (CI). One-sided t-test; CI shown symmetrically for visualization. Negative values indicate better (higher) rankings by the minimum-based strategy. Square dots represent results from the lenient drug target set (genes supported by ≥ 2 datasets), and circles from the moderate set (≥ 3 datasets). Dot colors indicate significance of the difference: blue for nominal ($p < 0.05$), red for Bonferroni-significant ($p < 0.05/60 = 8.3 \times 10^{-4}$). Diseases are grouped by ICD-10 categories, shown in the right boxes.

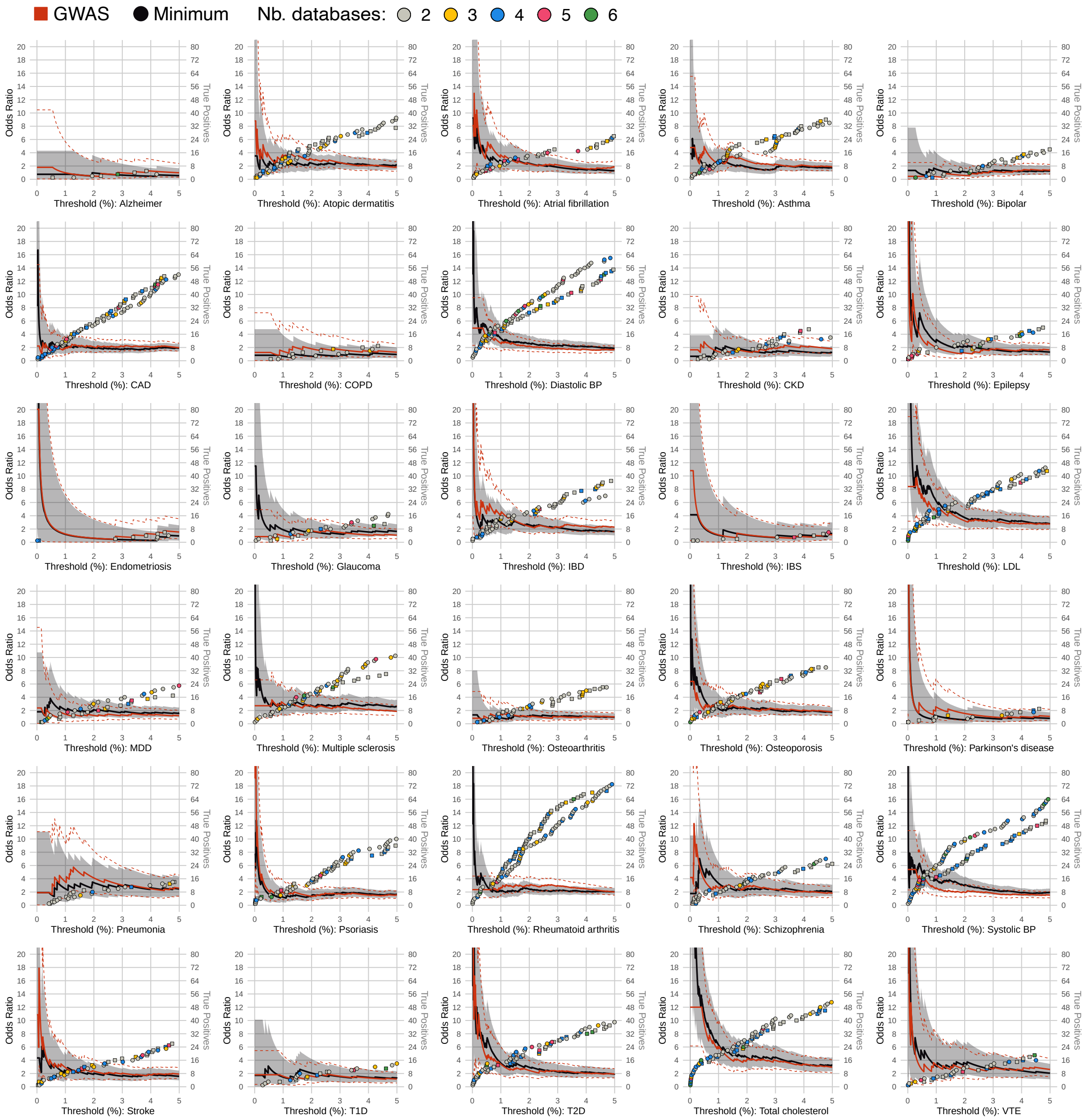

Fig. S4: Recovery of known drug targets in the lenient set across top five percentiles. Odds ratios (ORs) for recovering known drug targets among top-ranked genes in the 30 diseases across percentile thresholds (x-axis), based on the lenient drug target set (i.e., in ≥ 2 datasets). ORs are shown for the minimum-based (black) and GWAS (red) approaches; shaded areas and dotted lines indicate 95% confidence intervals (CI). Dots represent newly recovered true positive drug targets per threshold (circles: minimum, squares: GWAS), with cumulative counts on the right y-axis. Dot colors reflect the number of supporting datasets: grey (2), yellow (3), blue (4), magenta (5), and green (6). ORs are based on the lenient drug target definition. The left y-axis is truncated at OR = 20.

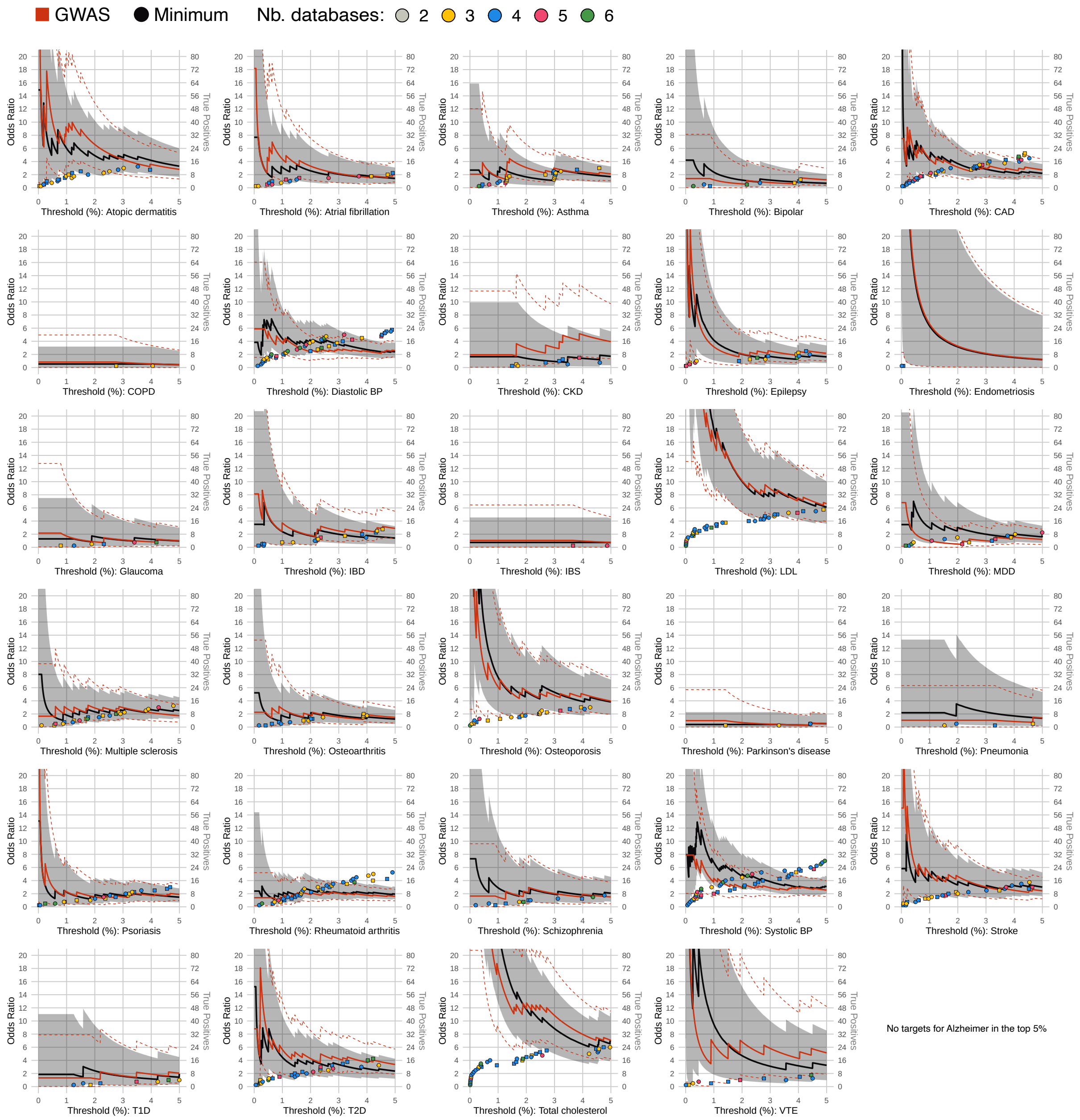

Fig. S5: Recovery of known drug targets in the moderate set across top five percentiles. Odds ratios (ORs) for recovering known drug targets among top-ranked genes in 29 diseases across percentile thresholds (x-axis, no targets found in top 5% for Alzheimer's disease), based on the moderate drug target set (i.e., in ≥ 3 datasets). ORs are shown for the minimum-based (black) and GWAS (red) approaches; shaded areas and dotted lines indicate 95% confidence intervals (CI). Dots represent newly recovered true positive drug targets per threshold (circles: minimum, squares: GWAS), with cumulative counts on the right y-axis. Dot colors reflect the number of supporting datasets: yellow (3), blue (4), magenta (5), and green (6). ORs are based on the moderate drug target definition. The left y-axis is truncated at OR = 20.

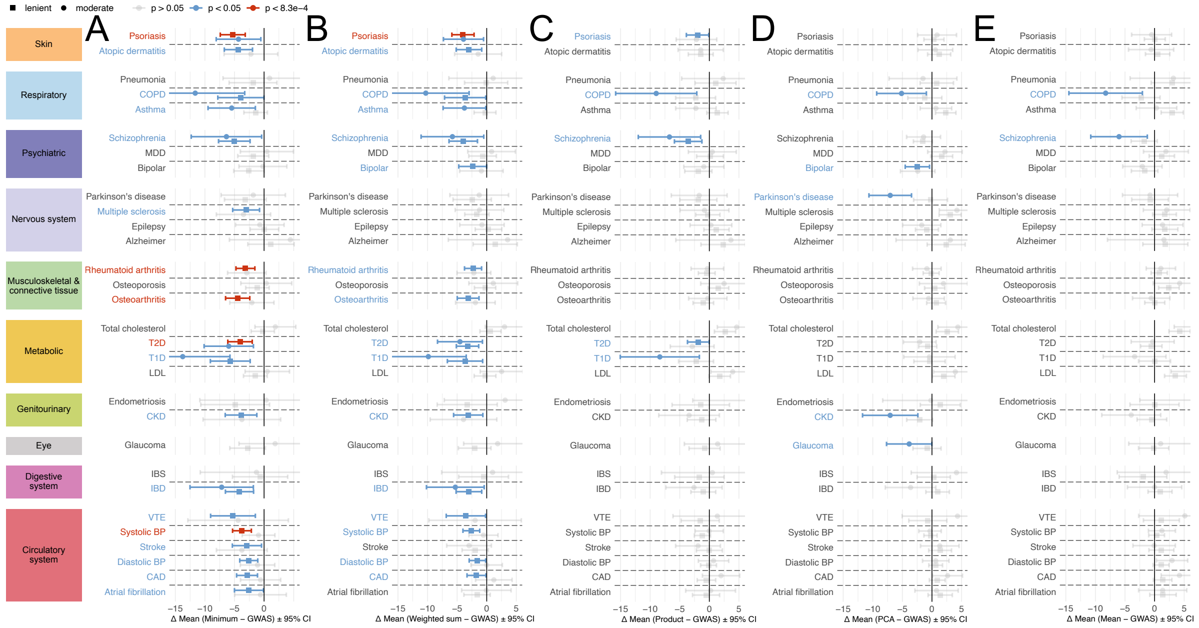

Fig. S6: Comparing integration strategies to GWAS gene prioritization for drug target identification. Mean difference between gene prioritization percentiles using the **(A)** minimum-based, **(B)** weighted sum-based, **(C)** product-based, **(D)** PCA-based, **(E)** average-based versus standard GWAS approach, across drug target genes within the GWAS gene space. The x-axis shows the mean difference (e.g., minimum − GWAS) with 95% confidence intervals (CI). One-sided t-test; CI shown symmetrically for visualization. Negative values indicate better (higher) rankings by the respective integration strategy compared to GWAS. Square dots represent results from the lenient drug target set (genes supported by ≥ 2 datasets), and circles from the moderate set (≥ 3 datasets). Dot colors indicate significance of the difference: grey for non-significant, blue for nominal ($p < 0.05$), red for Bonferroni-significant ($p < 0.05/60 = 8.3 \times 10^{-4}$). Diseases are grouped by ICD-10 categories, shown in the left-aligned boxes.

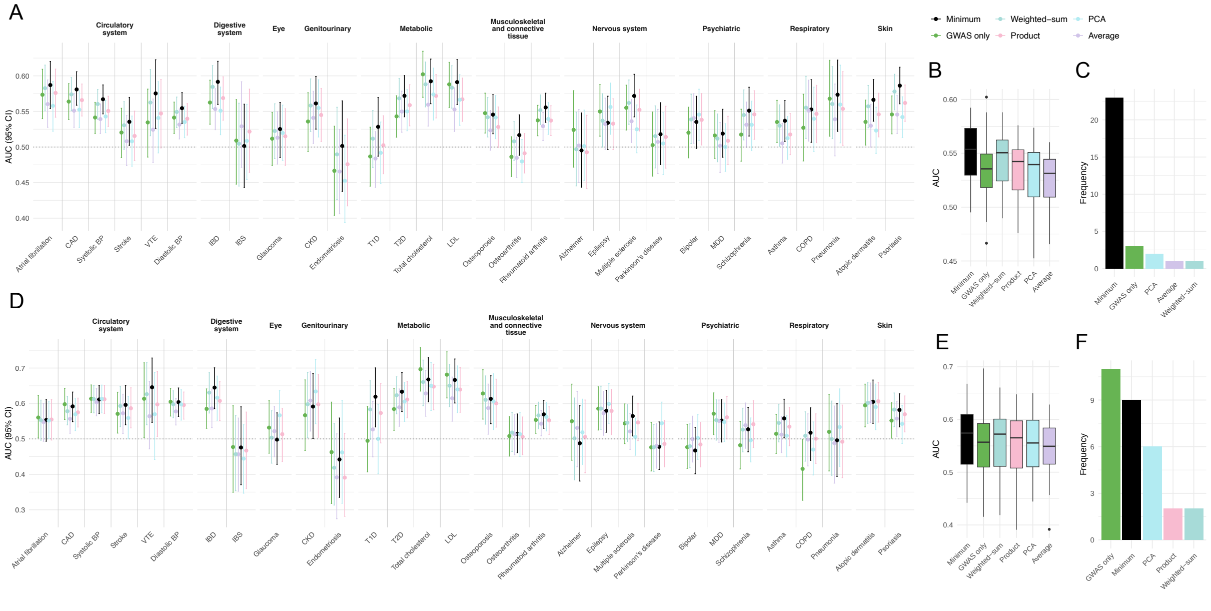

Fig. S7: AUROC performance comparison across strategies and drug target sets. **(A)** AUROC with 95% confidence intervals (CI) grouped by ICD-10 classes for the lenient drug target set (i.e., in ≥ 2 datasets). Approaches include: minimum-based (black), GWAS only (green), weighted-sum (pale green), product (pink), PCA (light blue), and average (light purple). **(B)** Boxplot showing AUROC distribution per strategy from Panel A. **(C)** Frequency plot showing how often each strategy achieved the highest AUROC across 30 diseases. **(D-F)** Same as Panels A–C, but for the moderate drug target set (i.e., in ≥ 3 datasets).

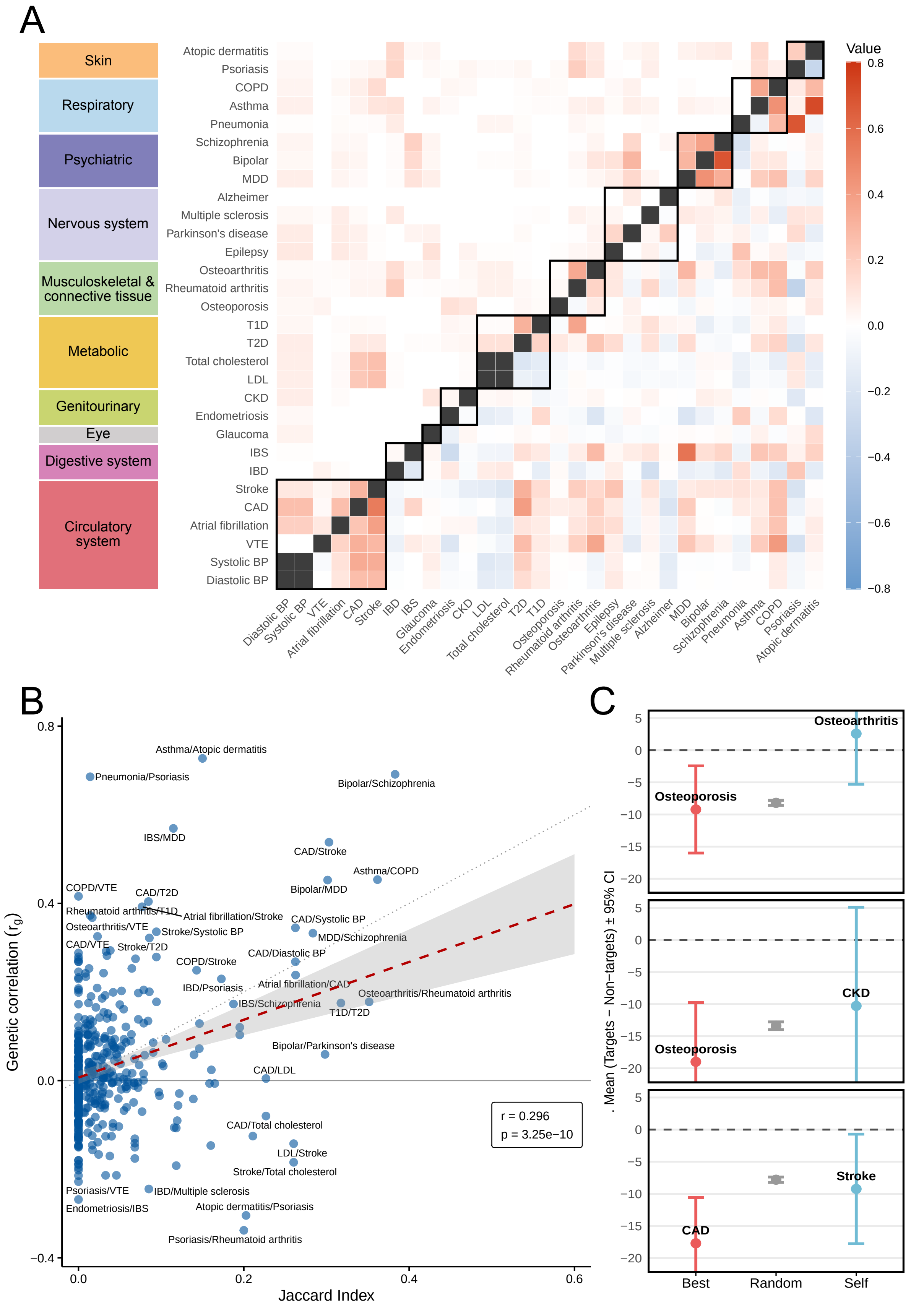

Fig. S8: Drug target overlap and cross-trait prioritization without VIP genes. **(A)** Heatmap comparing drug target genes overlap (upper triangle) and genetic correlation (lower triangle) between disease pairs.

Fig. S8: Drug target overlap and cross-trait prioritization without VIP genes (continued). Jaccard indices quantify the overlap of drug target genes from the moderate set (supported by ≥ 3 datasets) after removing very important pharmacogenes (VIP) genes (Additional file 1: Table S2). Genetic correlations (r_g) were computed using LDSC based on GWAS summary statistics. Positive r_g values are shown in red, negative in blue, and Jaccard indices are shaded according to the scale on the right. Diseases are grouped by ICD-10 categories, indicated on the left y-axis and emphasized with black borders along the diagonal. **(B)** Scatter plot comparing genetic correlation (y-axis) to drug target overlap (Jaccard index, x-axis) for all disease pairs (blue circles). A red dashed line represents the linear regression fit with 95% confidence interval in grey shading. The dotted diagonal is the identity line, and the solid horizontal grey line marks zero genetic correlation. Regression estimates are displayed in the bottom-right box. **(C)** Illustrative examples of cross-trait prediction of drug targets. For three diseases — osteoarthritis (top), chronic kidney disease (middle), and stroke (bottom) — we compare the mean percentile difference between drug targets and non-targets based on the minimum-based strategy. More negative values indicate better prioritization of true drug targets. The x-axis shows three predictor conditions: the best-performing disease ("Best", red dot), a random prioritization ("Random", grey dot; see Methods), and the disease itself ("Self", blue dot). Error bars show 95% confidence intervals. The dotted horizontal line at zero indicates no difference in mean percentiles between targets and non-targets.

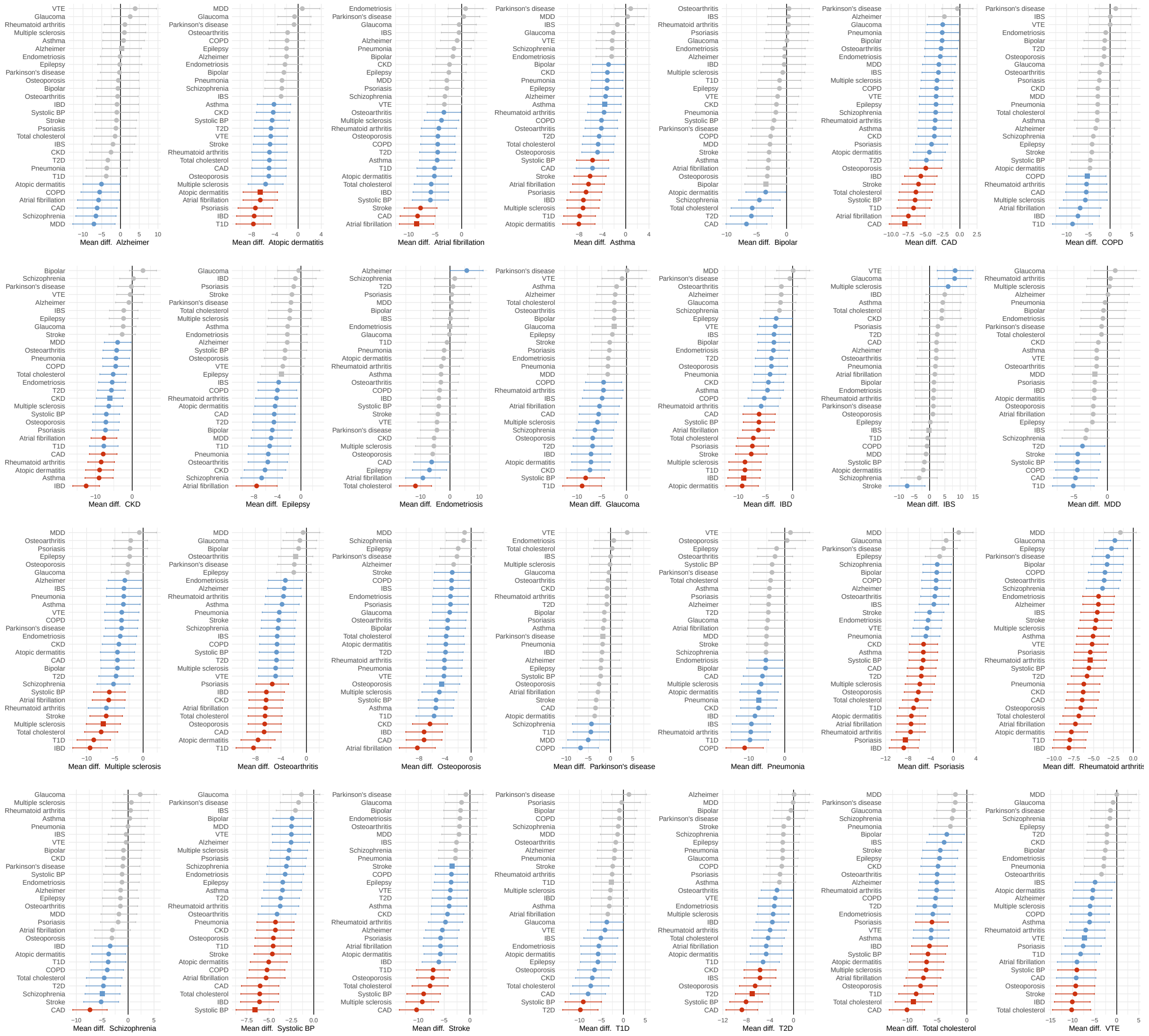

Fig. S9: Cross-trait drug target prediction from the lenient set (supported by ≥ 2 datasets). Cross-trait prediction of drug targets across 28 diseases, comparing the mean percentile difference between drug targets and non-targets using the minimum-based ranking across diseases (excluding LDL and DBP due to redundancy; x-axis). The x-axis shows the disease whose drug targets are evaluated (square dots), and the y-axis shows the disease used for prediction. More negative values indicate better prioritization of true drug targets. Dot color indicates significance from null: grey for non-significant, blue for $p < 0.05$, and red for Bonferroni significance ($p < 6.4e{-5}$).

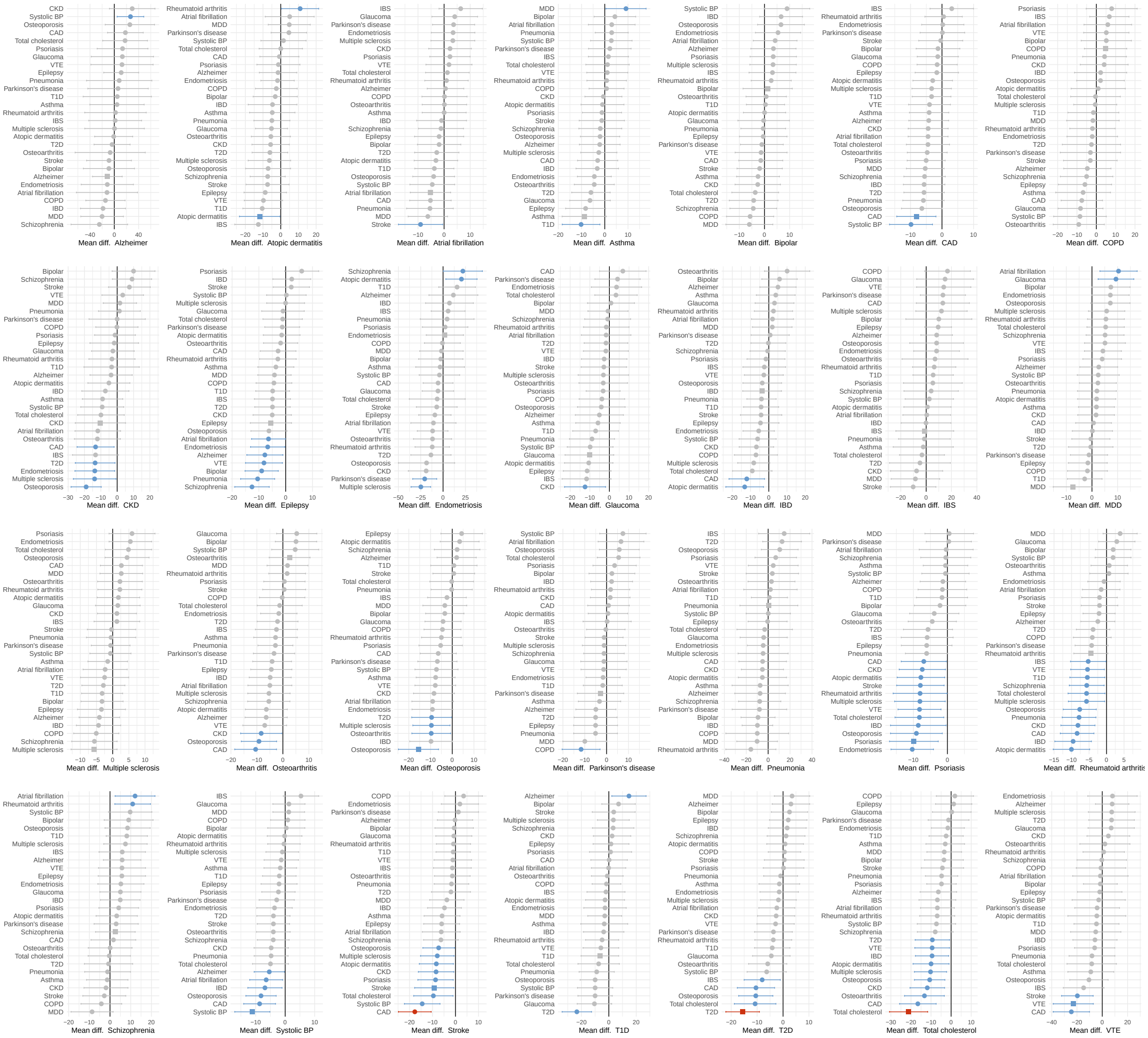

Fig. S10: Cross-trait drug target prediction from the moderate set (supported by ≥3 datasets) without VIP genes. Cross-trait prediction of drug targets across 28 diseases, comparing the mean percentile difference between drug targets and non-targets using the minimum-based ranking across diseases (excluding LDL and DBP due to redundancy; x-axis) without very important pharmacogenes (VIP, see Additional file 1: Table S2). The x-axis shows the disease whose drug targets are evaluated (square dots), and the y-axis shows the disease used for prediction. More negative values indicate better prioritization of true drug targets. Dot color indicates significance from null: grey for non-significant, blue for $p < 0.05$, and red for Bonferroni significance ($p < 6.4e{-}5$).

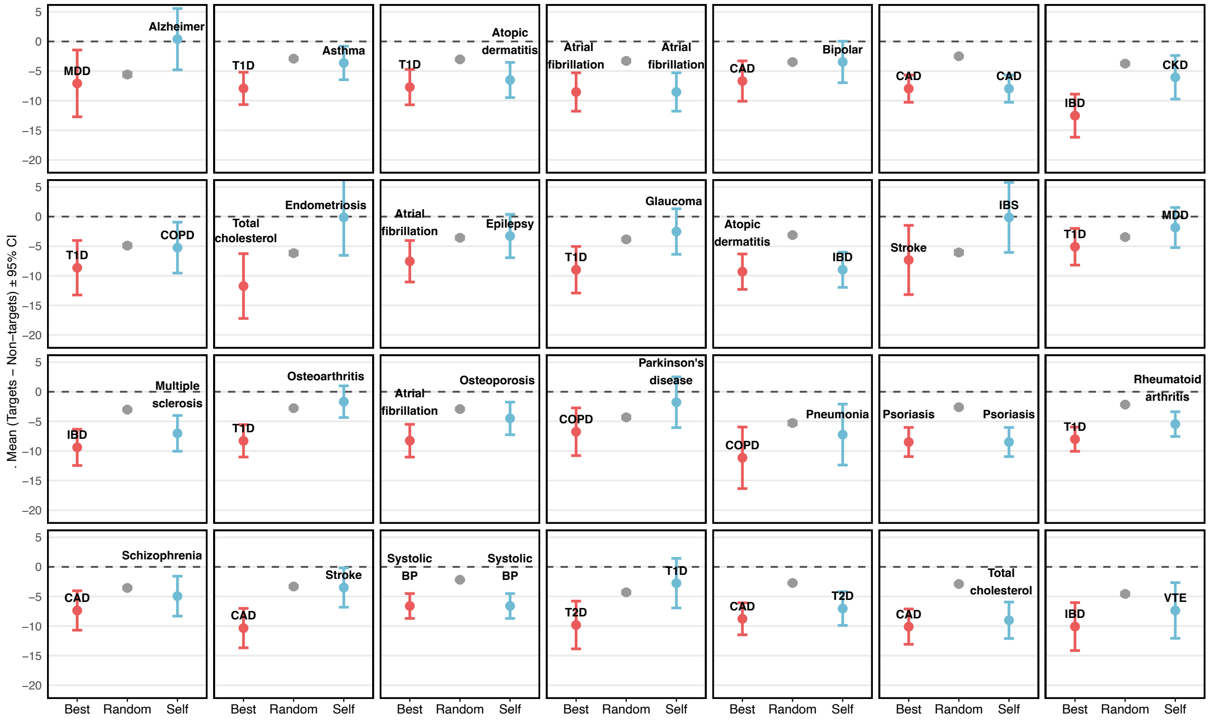

Fig. S11: Best cross-trait prediction of drug targets with random baseline comparison from the lenient set. Cross-trait prediction of drug targets across 28 diseases (excluding LDL and DBP due to redundancy), comparing the mean percentile difference between targets and non-targets using the minimum-based ranking across diseases. More negative values indicate better prioritization of true drug targets. The x-axis shows three predictor conditions: the best-performing disease ("Best", red dot), a random prioritization ("Random", grey dot; see Methods), and the disease itself ("Self", blue dot). Error bars show 95% confidence intervals. The dotted horizontal line at zero indicates no difference in mean percentiles between targets and non-targets.

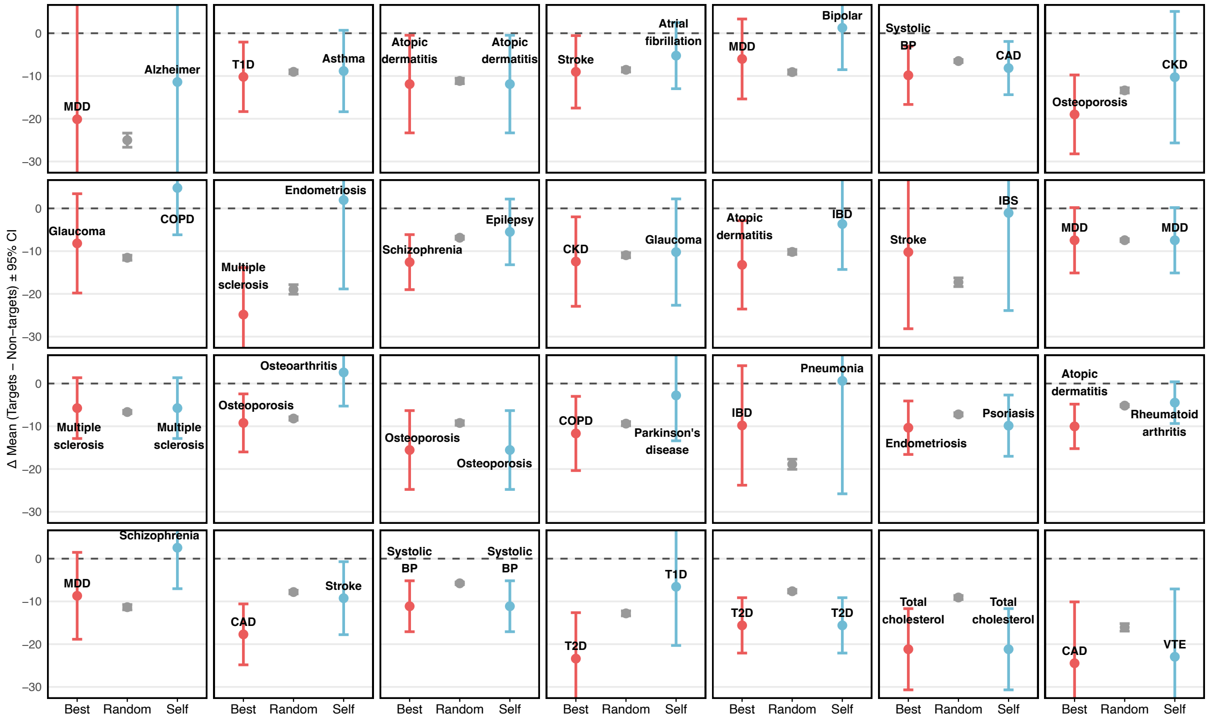

Fig. S12: Best cross-trait prediction of drug targets with random baseline comparison from the moderate set without VIP genes. Cross-trait prediction of drug targets across 28 diseases (excluding LDL and DBP due to redundancy), comparing the mean percentile difference between targets and non-targets using the minimum-based ranking across diseases without very important pharmacogenes (VIP). More negative values indicate better prioritization of true drug targets. The x-axis shows three predictor conditions: the best-performing disease ("Best", red dot), a random prioritization ("Random", grey dot; see Methods), and the disease itself ("Self", blue dot). Error bars show 95% confidence intervals. The dotted horizontal line at zero indicates no difference in mean percentiles between targets and non-targets.

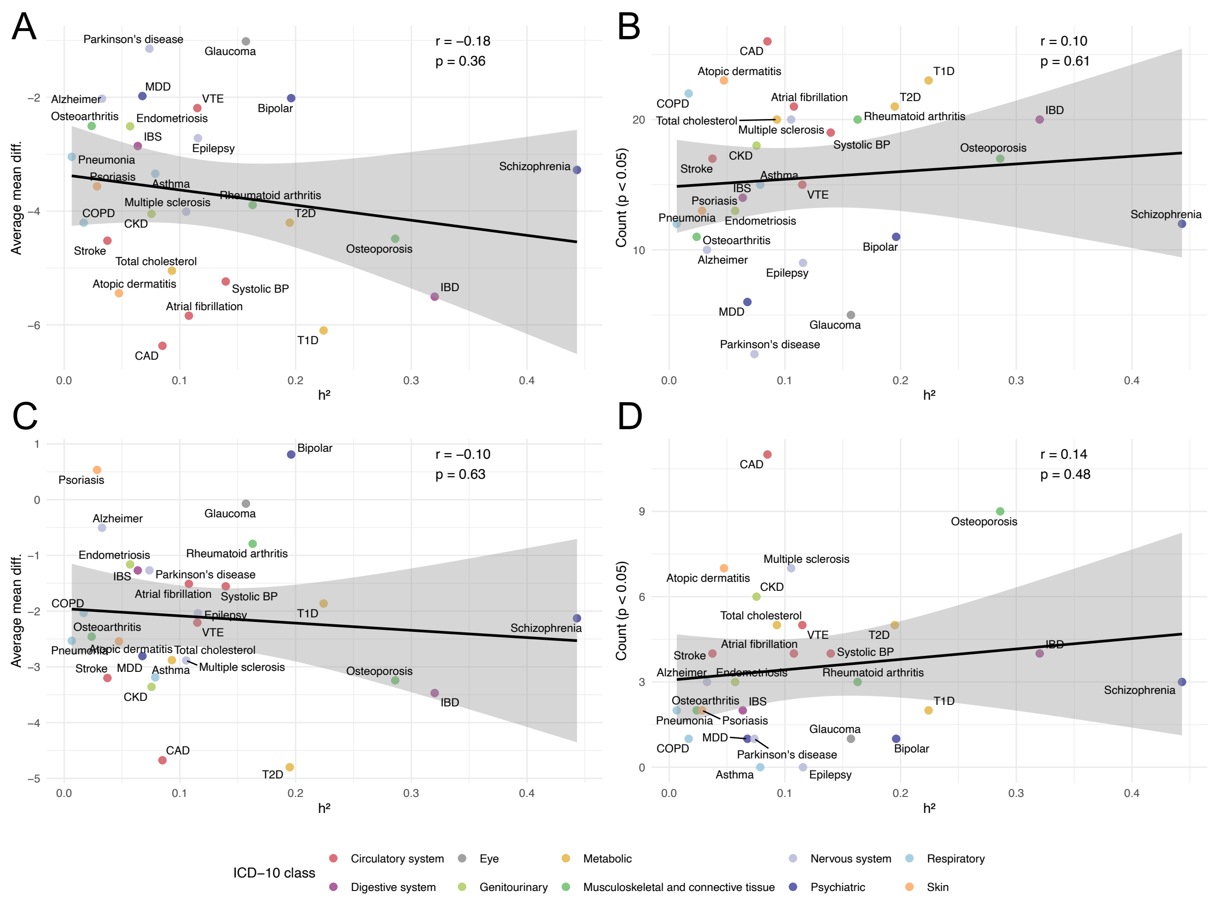

Fig. S13: Relationship between SNP-heritability and cross-trait target prioritization performance. Each point represents a trait used as a scoring trait for drug target prioritization across diseases. Panels **A**–**B** correspond to the lenient configuration, whereas **C**–**D** correspond to the moderate configuration without VIP genes. The x-axis shows SNP-heritability estimates (h^2; see Methods). In panels **A and C**, the y-axis shows the average mean difference in ranking between drug targets and non-target genes for the focal trait, averaged across all traits evaluated. In panels **B and D**, the y-axis shows the number of traits for which the focal trait nominally significantly ranks drug targets above non-target genes ($p < 0.05$; see Additional file 2: Figs. S9-S10). The black line shows the linear regression fit with 95% confidence interval in grey shading. Pearson correlation coefficients (r) and corresponding p-values are shown in the upper right corner of each panel. Points are colored according to ICD-10 disease class.

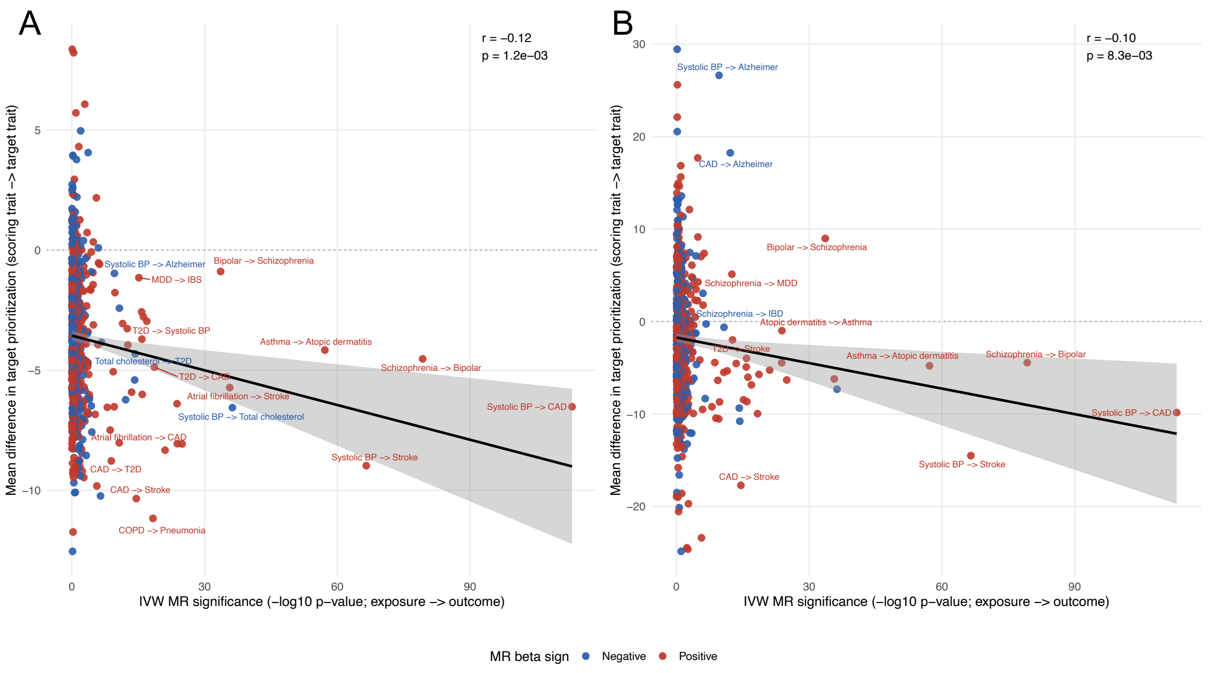

Fig. S14: Relationship between MR evidence across traits and cross-trait target prioritization performance. Pairwise trait relationships were evaluated by comparing Mendelian randomization (MR) evidence (see Additional file 1: Table S7) with cross-trait target prioritization performance. The x-axis shows the significance of the MR association between traits ($-log_{10}$ p-value from inverse-variance weighted MR), corresponding to the effect of the exposure trait on the outcome trait (with some pairs labeled in the format $exposure \rightarrow outcome$). The y-axis shows the improvement in cross-trait target prioritization, defined as the mean difference in ranking between drug targets and non-target genes when one trait is used to score genes and targets of another trait are evaluated ($scoring\ trait \rightarrow evaluated\ trait$). Panels correspond to the **(A)** lenient and **(B)** moderate configurations without VIP genes. Points are colored according to the sign of the MR causal estimate (positive in red and negative in blue). The black line shows the linear regression fit with 95% confidence interval in grey shading. Pearson correlation coefficients (r) and corresponding p-values are shown in the upper right corner of each panel.

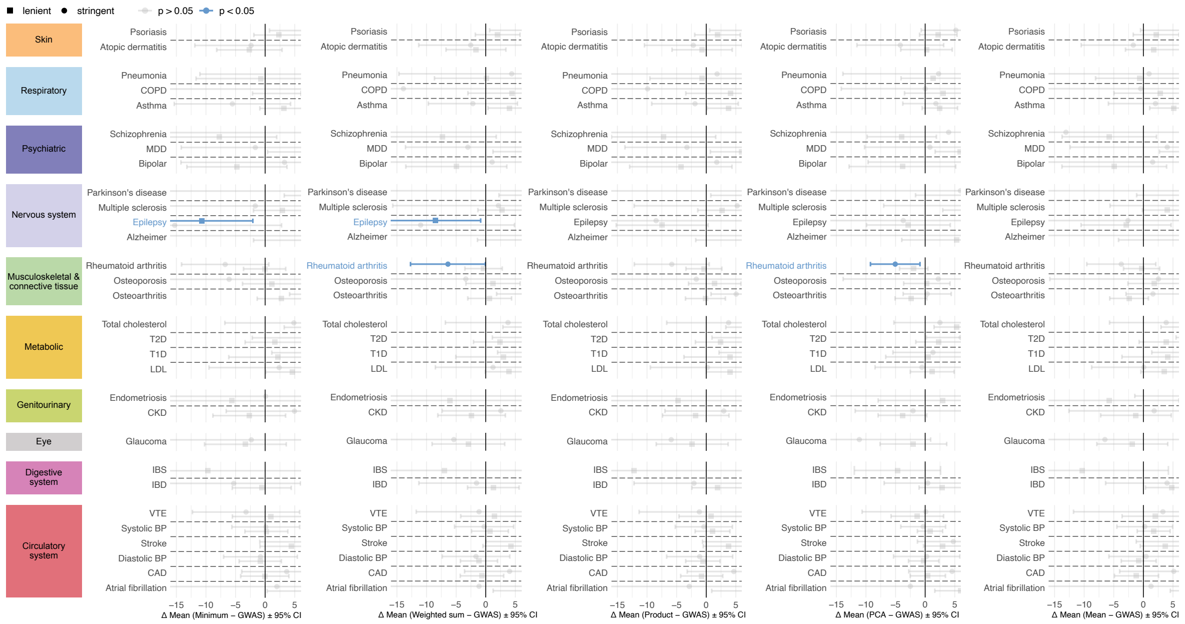

Fig. S15: Comparing integration strategies to GWAS gene prioritization for drug target identification on complete data. Mean difference between gene prioritization percentiles using the **(A)** minimum-based, **(B)** weighted sum-based, **(C)** product-based, **(D)** PCA-based, **(E)** average-based versus standard GWAS approach, across drug target genes within the GWAS gene space. The x-axis shows the mean difference (e.g., minimum − GWAS) with 95% confidence intervals (CI). One-sided t-test; CI shown symmetrically for visualization. Negative values indicate better (higher) rankings by the respective integration strategy compared to GWAS. Square dots represent results from the lenient drug target set (genes supported by ≥ 2 datasets), and circles from the moderate set (≥ 3 datasets). Dot colors indicate significance of the difference: grey for non-significant, blue for nominal ($p < 0.05$). Diseases are grouped by ICD-10 categories, shown in the left-aligned boxes.

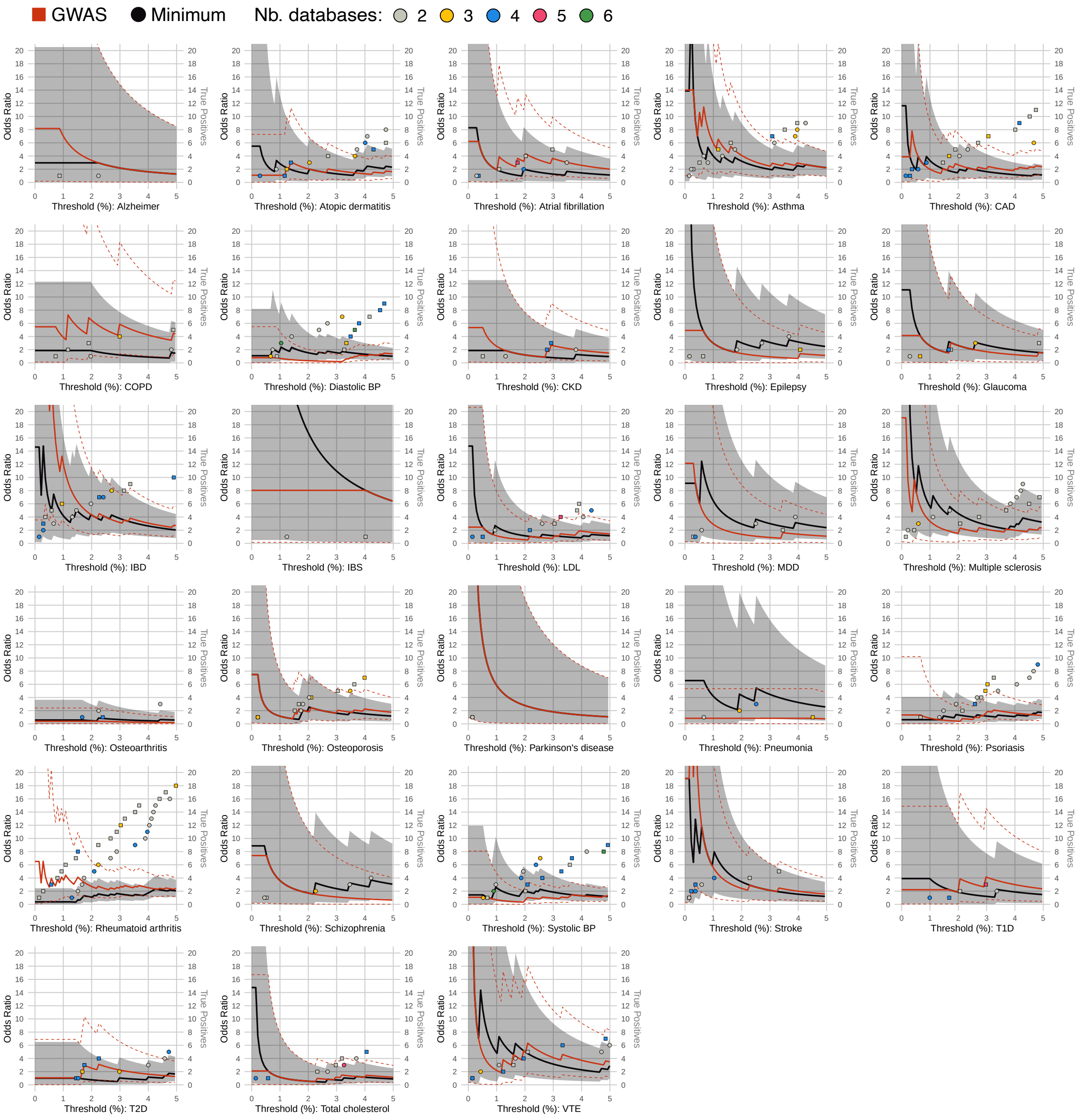

Fig. S16: Recovery of known drug targets in the lenient set across top five percentiles on complete data. Odds ratios (ORs) for recovering known drug targets among top-ranked genes in 28 diseases with identified targets in the top 5% across percentile thresholds (x-axis), based on the lenient drug target set (i.e., in ≥ 2 datasets). ORs are shown for the minimum-based (black) and GWAS (red) approaches; shaded areas and dotted lines indicate 95% confidence intervals (CI). Dots represent newly recovered true positive drug targets per threshold (circles: minimum, squares: GWAS), with cumulative counts on the right y-axis. Dot colors reflect the number of supporting datasets: grey (2), yellow (3), blue (4), magenta (5), and green (6). ORs are based on the lenient drug target definition. The left y-axis is truncated at OR = 20.

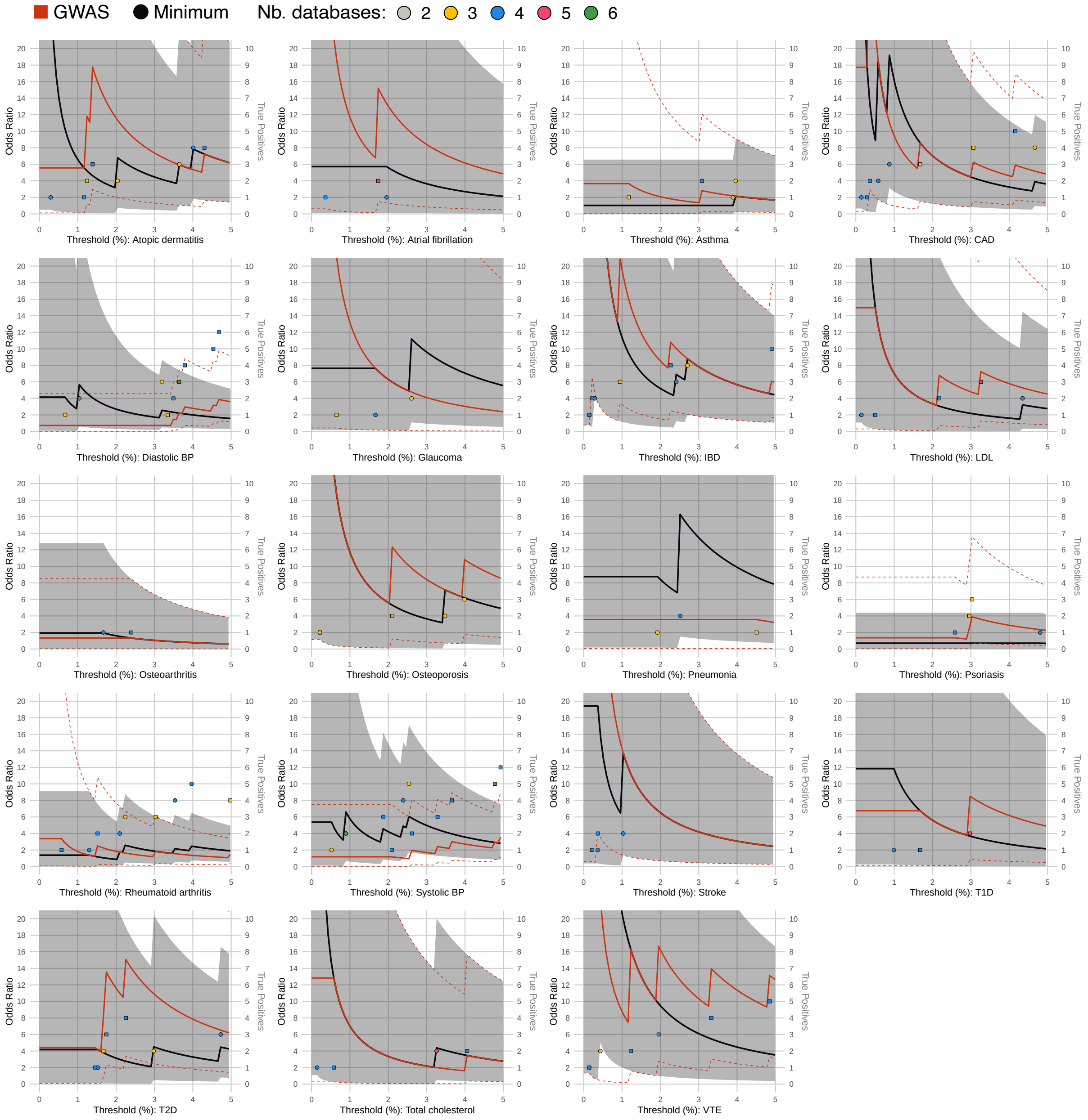

Fig. S17: Recovery of known drug targets in the moderate set across top five percentiles on complete data. Odds ratios (ORs) for recovering known drug targets among top-ranked genes in 19 diseases with identified targets in the top 5% across percentile thresholds (x-axis), based on the moderate drug target set (i.e., in ≥ 3 datasets). ORs are shown for the minimum-based (black) and GWAS (red) approaches; shaded areas and dotted lines indicate 95% confidence intervals (CI). Dots represent newly recovered true positive drug targets per threshold (circles: minimum, squares: GWAS), with cumulative counts on the right y-axis. Dot colors reflect the number of supporting datasets: yellow (3), blue (4), magenta (5), and green (6). ORs are based on the moderate drug target definition. The left y-axis is truncated at OR = 20.

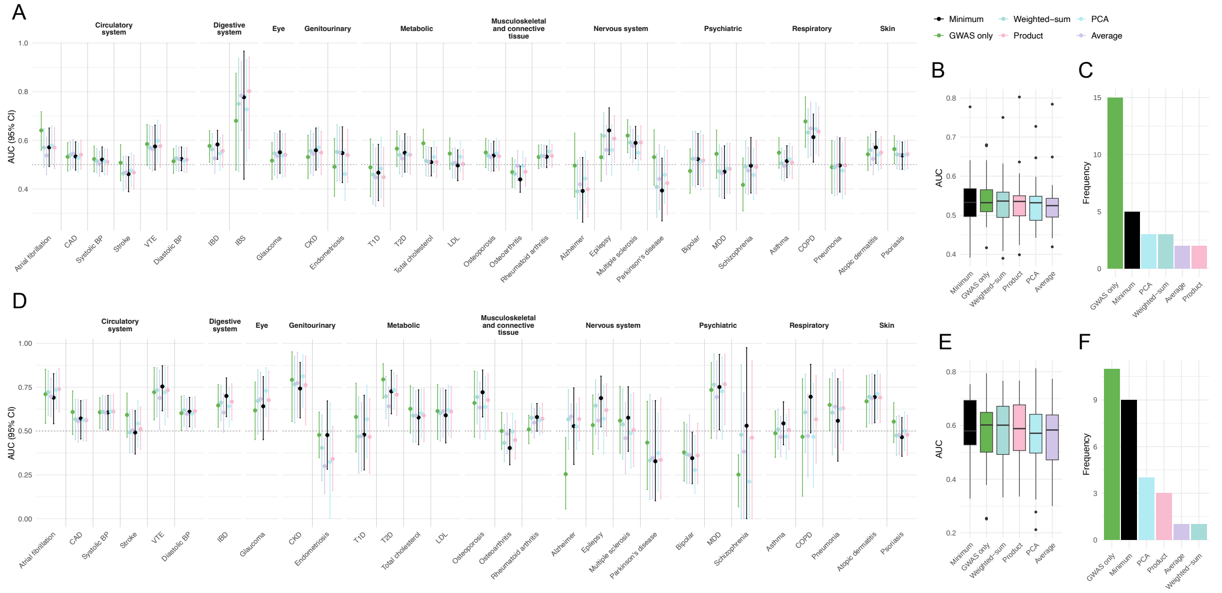

Fig. S18: AUROC performance comparison across strategies and drug target sets on complete data. **(A)** AUROC with 95% confidence intervals (CI) grouped by ICD-10 classes for the lenient drug target set (i.e., in ≥ 2 datasets). Approaches include: minimum-based (black), GWAS only (green), weighted-sum (pale green), product (pink), PCA (light blue), and average (light purple). **(B)** Boxplot showing AUROC distribution per strategy from Panel A. **(C)** Frequency plot showing how often each strategy achieved the highest AUROC across 30 diseases. **(D-F)** Same as Panels A–C, but for the moderate drug target set (i.e., in ≥ 3 datasets).

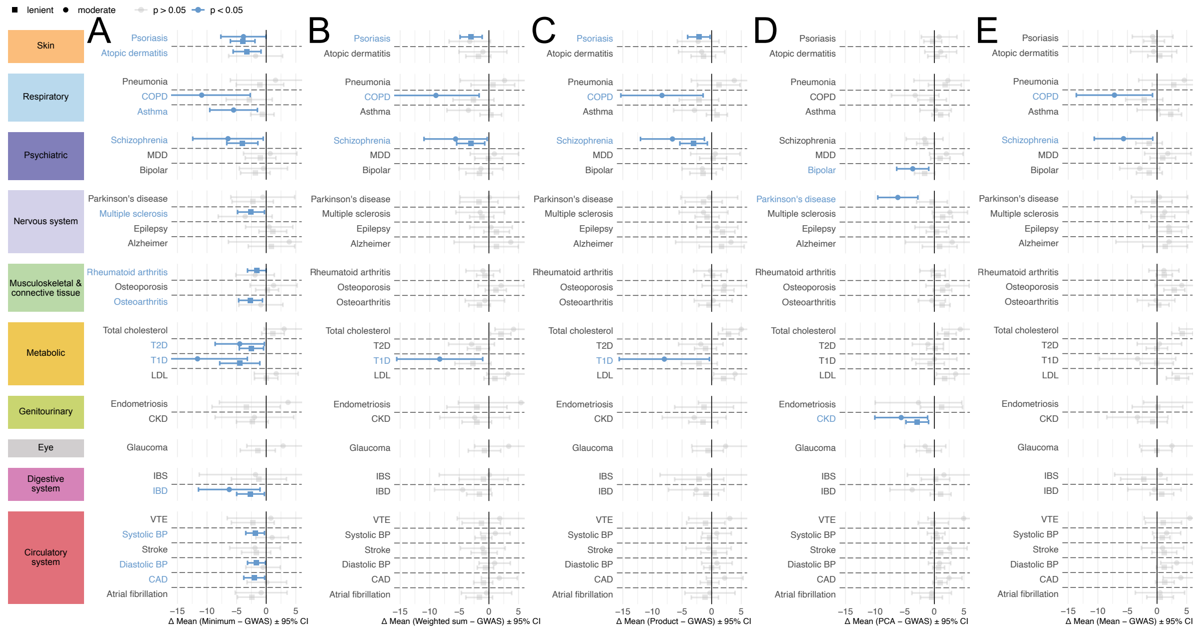

Fig. S19: Comparing integration strategies to GWAS gene prioritization for drug target identification without pQTL results. Mean difference between gene prioritization percentiles using the **(A)** minimum-based, **(B)** weighted sum-based, **(C)** product-based, **(D)** PCA-based, **(E)** average-based without pQTL data versus standard GWAS approach, across drug target genes within the GWAS gene space. The x-axis shows the mean difference (e.g., minimum − GWAS) with 95% confidence intervals (CI). One-sided t-test; CI shown symmetrically for visualization. Negative values indicate better (higher) rankings by the respective integration strategy compared to GWAS. Square dots represent results from the lenient drug target set (genes supported by ≥ 2 datasets), and circles from the moderate set (≥ 3 datasets). Dot colors indicate significance of the difference: grey for non-significant, blue for nominal ($p < 0.05$). Diseases are grouped by ICD-10 categories, shown in the left-aligned boxes.
